# Physicochemical, nutritional and biological characterization of different Indian monofloral honeys with special emphasis on their glucose-lowering efficacy

**DOI:** 10.1016/j.fochx.2026.104198

**Published:** 2026-07-17

**Authors:** Chandrachur Ghosh, Somsuvra Chatterjee, Tiyasa Pathak, Tathagata Kundu, Surendra Saini, Debabrata Sircar, Prabhat Kumar, Partha Roy

**Affiliations:** a Molecular Endocrinology Laboratory, Department of Biosciences and Bioengineering, Indian Institute of Technology Roorkee, Roorkee 247 667, Uttarakhand, India; b Plant Molecular Biology Laboratory, Department of Biosciences and Bioengineering, Indian Institute of Technology Roorkee, Roorkee 247 667, Uttarakhand, India; c National Bee Board, DA & FW, Ministry of Agriculture and Farmers Welfare, B Wing, 2nd Floor, Janpath Bhawan, Janpath, New Delhi 110 001, India; d Center for Indian Knowledge Systems, Indian Institute of Technology Roorkee, Roorkee 247 667, Uttarakhand, India

**Keywords:** Indian honey, Biophysical characterization, Nutritional characterization, Glucose-lowering efficacy

## Abstract

It is important to provide consumers with pure and natural honey. Toward this end, six biophysical, eight nutritional, and two antioxidant activity-related parameters were investigated in four different Indian monofloral honey samples using various analytical techniques, in accordance with globally accepted standards and using manuka honey for certain parameters. The honey samples were also subjected to their *in vitro* glucose-lowering efficacy in palmitic acid-challenged insulin-resistant HepG2 cell model. The results showed that in all the honey samples as tested here, hydroxymethylfurfural (HMF), free acidity, and ash levels complied with the criteria of standard pure honey. Total of twenty-two different polyphenols and twenty-four different terpenoids were found in these honey samples, although the predominant bioactive compound varied between these honey samples. Some of these honey samples also showed efficient glucose-lowering activity. Overall, the study provides a thorough insight regarding the physicochemical, nutritional and biological properties of different varieties of Indian honeys.

## Highlights

• This study validates the quality of four Indian unifloral honey and evaluates their various biophysical and biochemical characteristics.
• Various polyphenols and terpenoids were identified and compared among each sample for their geographical and botanical origin.
• This study determines the inhibitory efficiency as well as the type of inhibition of all the honey samples against α-amylase and α-glucosidase enzymes.
• Various cell-based assays demonstrate the glucose-lowering and lipid-dissolving efficiency of CiH and LyH.

## Graphical abstract

**Figure:**
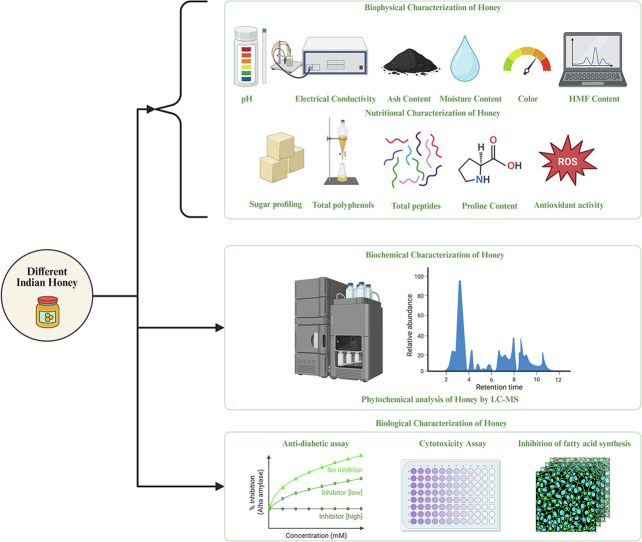

## 1 Introduction

Honey has long been recognized for its therapeutic value, yet the biological basis underlying these effects, particularly its emerging glucose-modulatory potential, remains insufficiently defined. Although numerous studies have investigated various honeys for their biological efficacies, to the best of our knowledge, this work represents the first comprehensive profiling specifically targeting Indian monofloral honeys with respect to their putative antidiabetic or glucose-lowering activity. While previous studies largely focused on individual honey varieties or general phenolic content correlations, current study uniquely integrates extensive physicochemical, nutritional, and functional analyses across multiple Indian honey types, linking distinct biochemical compositions directly to glucose-lowering mechanisms. Moreover, unlike most regional and international honey characterization studies, which primarily document compositional attributes without establishing mechanistic links, this study advances the field by coupling detailed biochemical profiling with functional antidiabetic outcomes, thereby offering a level of integrative analysis not previously achieved in global honey research. This focused evaluation of Indian honey’s antidiabetic potential provides novel mechanistic insights within a regional but globally relevant context, addressing a previously unexplored gap in both the biochemical characterization and biological evaluation of these honeys. Although honey contains diverse sugars, phytochemicals, amino acids, minerals, and enzymes (Akbari et al., 2020; Azonwade et al., 2018; Crăciun et al., 2020), existing literature largely catalogues these constituents without clarifying how specific components relate to measurable bioactivity. Many previous studies describing antibacterial (Weston, 2000), anti-inflammatory (Kassim et al., 2010), cardioprotective (Khalil et al., 2015), hepatoprotective (Erejuwa et al., 2011), and antioxidant effects (Beretta et al., 2005) highlight honey’s therapeutic breadth, but the mechanistic determinants of these outcomes are not well established. While several studies have examined phenolics and antidiabetic activity in monofloral honeys, they generally focus on correlational profiles rather than identifying which distinct phytochemical or physicochemical features causally underpin glucose-lowering actions. Recent findings that certain honey varieties exhibit lower glycemic impact than equivalent sugar loads (Abdulrhman et al., 2013; Erejuwa et al., 2012), and that honey and equivalent sugar intake can produce contrasting inflammatory profiles (Fakhlaei et al., 2020; Ghosh et al., 2025; Zulkifli et al., 2020) reinforce that compositional differences may translate into biologically meaningful effects, yet the specific attributes driving this remain unknown.

This knowledge gap is particularly important given the increasing prevalence of adulterated honey, where elevated fructose content can exacerbate metabolic dysregulation, including increased serum uric acid, insulin resistance, non alcoholic fatty liver disease (NAFLD) risk, hepatic lipogenesis, and tissue injury (Leone et al., 2021). Such contrasting outcomes between authentic and adulterated honey underscore the need to pinpoint which compositional factors, beyond simple sugar content, drive beneficial versus harmful metabolic responses. Although honey quality parameters such as sugars, phytochemicals, proline, minerals, enzymes, and HMF are well established (Abdel-Aal et al., 1993; Khan et al., 2024; Meyers et al., 2017), these indicators alone do not explain functional variability, and current regulatory frameworks including the European Community Guidelines 2001/110 emphasize physicochemical standards without linking them to biological efficacy (Truong et al., 2024). Thus, a clear gap remains in integrating chemical characterization with functional outcomes in a mechanistic manner, an aspect not resolved in existing regional or international honey characterization studies.

Indian monofloral honeys provide a model system to interrogate this relationship because of their diverse and distinct botanical origins and corresponding phytochemical signatures. Mustard honey (MuH), enriched with glucosinolate-derived metabolites and unique terpenoids such as globulol, has been associated with hypoglycemic potential, whereas manuka honey, despite its methylglyoxal-mediated antimicrobial activity (Angane et al., 2024; Johnston et al., 2018), lacks comparable anti-diabetic phytochemical (Dey & Mitra, 2013; Garg et al., 2023; Usman et al., 2022). Globulol is notably absent in manuka plant (*Leptospermum scoparium*) tissues (Hoang & Van Vuong, 2025), whereas trace amounts occur in related *Leptospermum* species (*L. pallidum* and *L.* *madidum* contain a small amount of this terpenoid in their leaves). These contrasting phytochemical profiles allow this study to move beyond prior compositional surveys by directly linking detailed biochemical characterization with functional glucose-regulatory assessment, thereby addressing the unresolved question of how specific honey components mechanistically contribute to antidiabetic efficacy. In this study, four different types of Indian unifloral honey (cinnamon (CiH), coconut (CoH), lychee (LyH) and mustard (MuH) honey) were collected from different regions of India and screened for their various physicochemical and nutritional parameters. Next, the *in vitro* glucose-lowering assays (cell-free enzymatic as well as cell-based) were performed to test the anti-diabetic efficacy of these Indian honey samples. While prior studies have characterized phenolics and antidiabetic potential in monofloral honeys from various regions, they primarily reported compositional surveys without establishing mechanistic links between cultivar-specific phytochemical signatures and functional glucose-lowering outcomes. This study, to the best of our knowledge, addresses this critical gap by providing the first integrated profiling of Indian monofloral honeys (CiH, CoH, LyH, MuH), combining SEM-authenticated melissopalynology, LC-MS fingerprinting (22 polyphenols, 24 terpenoids), and functional validation through α-amylase/α-glucosidase inhibition kinetics alongside insulin sensitization in palmitic acid-induced HepG2 steatosis models. By directly correlating distinctive polyphenol-terpenoid profiles (e.g., pinocembrin dominance in MuH/LyH) with demonstrated enzymatic and cellular efficacy, this study advances beyond existing literature to enable predictive standardization of honey cultivars for therapeutic nutraceutical development. Taken together, this study represents a complete profile of four different types of Indian unifloral honey in terms of their physicochemical, nutritional and health-beneficial effects on regulating diabetes mellitus.

## 2 Materials and methodology

### 2.1 Sample collection

**Fig. 1:**
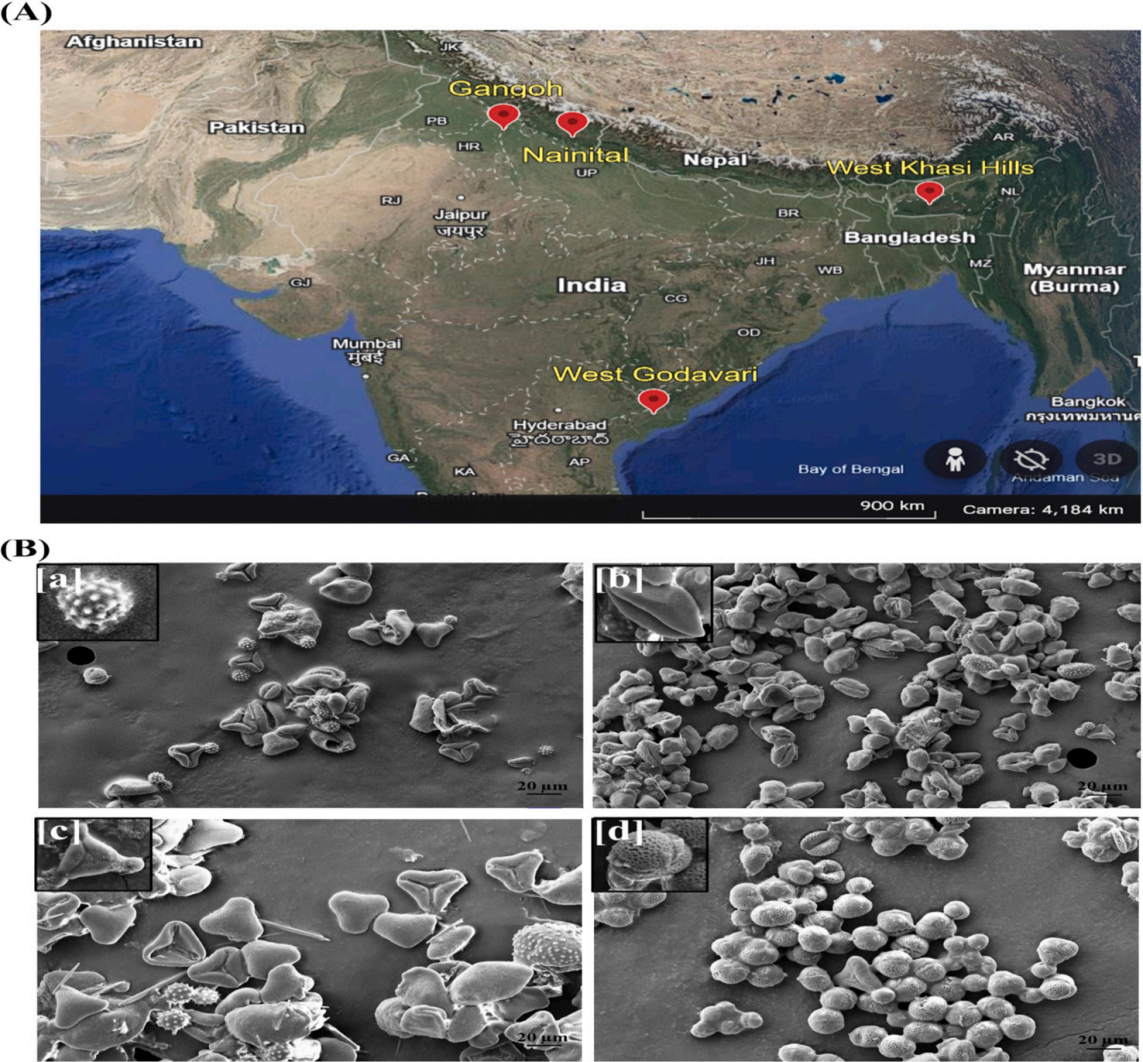
Collection and morphological analysis of different Indian monofloral honeys. [A] Representative map showing the geographical locations of the collection points of the honey samples used in this study (indicated by red dots). [B] Representative SEM images for pollen analysis of different Indian monofloral honeys: CiH [a], CoH [b], LyH [c], and MuH [d]. Scale bar: 20 μm and magnification: 500×. CiH, cinnamon honey; CoH, coconut honey; LyH, lychee honey; MuH, mustard honey. (For interpretation of the references to colour in this figure legend, the reader is referred to the web version of this article.)

Four Indian unifloral honeys were collected from local vendors in different parts of India. Lychee honey (from here onwards as LyH) (batch # JMF-109) was procured from Jyoti Gramodyog Sansthan (Regd.), Gangoh, Khadi, Uttar Pradesh, India. Cinnamon honey (CiH), Coconut honey (CoH) and Mustard honey (MuH) were collected from local farmers in West Khasi Hill district in Meghalaya, West Godavari district in Andhra Pradesh and Nainital district in Uttarakhand state in India, respectively. The geographical areas are marked red in Fig. 1A. All bioassays were performed using whole raw honey, expressed as mg/mL concentration, representing the weight of honey dissolved per mL of solvent/media. This approach was chosen to evaluate the biological activity of raw honey as a potential nutraceutical, since fractionation could lead to loss of synergistic effects among its native components.

### 2.2 Melissopalynological analysis

Honey samples (10 g each) were dissolved in distilled water (50 mL), centrifuged at 4000 rpm for 10 min at 4 °C (three consecutive cycles), and the final pellet was processed using the acetic anhydride method for acetolysis. Slides were prepared with glycerin jelly and examined under a microscope (Carl Zeiss, Oberkochen, Germany). A minimum of 500 pollen grains per sample were counted and identified to botanical family/genus/species level. Pollen frequency was calculated as % of total pollen, with uncountable pollen types excluded. Dominant (>45%), secondary (16-45%), minor (3-15%), and trace (<3%) pollen classes were classified as per international melissopalynological standards.

### 2.3 SEM imaging for pollen analysis

Pollen grains from four unifloral honey samples, CiH, CoH, LyH, and MuH, were extracted following the previously described protocol (Dhawan et al., 2018). Briefly, 1 g of each honey sample was dissolved in 2 mL of warm distilled water (40 °C) and centrifuged at 3000 ×*g* for 10 min. The supernatant was discarded, and the sediment was washed twice with distilled water. The remaining pollen pellet was dehydrated using an ethanol series (50%, 70%, 90%, and absolute ethanol) and air-dried on a glass slide, and then the samples were analyzed under Carl Zeiss Ultra Plus Field-Emission Scanning Electron Microscope (Germany).

### 2.4 Biophysical characterization

#### 2.4.1 pH measurement

For measuring the pH of honey, individual honey was dissolved in water to make a 20% (*w*/*v*) solution and then measured with a pH meter (Eutech pH/°C meter, Thermo Fischer Scientific, USA). Prior to the estimation, the pH meter was calibrated with standards (Aljohar et al., 2018).

#### 2.4.2 Electrical conductivity

The 20% (w/v) solutions of different honey were made with MilliQ water and then the electrical conductivity of different honey solutions was measured with a conductivity meter (Lab Junction, model- LJ-611, Kingston Lab Solutions, India) (Acquarone et al., 2007).

#### 2.4.3 Ash content

Using the principles of conductivity, ash content of respective honey solutions was calculated by applying the following formula according to the method described earlier (El Sohaimy et al., 2015)-.

$$\mathrm{Ash}\;\text{Content}\;\left(\%\right)=\left(0.083\times \text{Conductivity}\right)–0.092$$

#### 2.4.4 Moisture and total soluble solid content

The moisture and the total soluble solid content of different honey samples were measured using a refractometer (Erma, Tokyo, Japan) according to previously published protocols (Cano et al., 2001; Cereser Camara & Laux, 2010). The moisture content value was obtained by the refractive index at 25 °C and the total soluble solid materials was measured by the Brix value as per the protocol described earlier (AOAC, 1990).

#### 2.4.5 Colour of honey

The colour of different honey samples was measured by the Pfund scale as described previously (Bodor et al., 2021). For spectral analysis, the samples were diluted to 50% by distilled water (*w*/*v*). The spectral acquisition was performed in the 380-780 nm wavelength range at 25 °C in a spectrophotometer (BMG LabTech, FLUOstar OMEGA, Germany). For this assay, distilled water was used as blank. The formula used to convert the absorbance value was-

$$\text{Pfund}\;\left(\mathrm{mm}\right)=-38.70+371.39\times \mathrm{Abs}\;\left(635\;\mathrm{nm}\right)$$

#### 2.4.6 Hydroxymethylfurfural (HMF) content

Honey samples (1 g) were dissolved in approximately 5 mL Milli-Q water. Then 0.5 mL of a 15% (w/v) potassium hexacyanoferrate solution and 0.5 mL of a 30% (w/v) zinc acetate dehydrate solution were added, and 50 mL of Milli-Q water was used to make up the mixture to clear the honey samples and stop the HMF breakdown. The solution was then filtered by a 0.22 μm membrane filter before being injected into the HPLC, equipped with binary HPLC pump 1525 and PDA detector 2998 (Waters Corporation, Massachusetts, USA), for HMF measurement. A 25 cm × 4.6 mm Supelcosil LC-18, reverse phase stainless steel column (Supelco, Bellefonte, PA, USA) was used and operated at 30 °C. The mobile phase used was Water:methanol (90:10, v/v), and the flow rate was maintained at 0.75 mL/min throughout the run. In Milli-Q water, serial standard solutions of HMF (20-100 mg/mL) were prepared to produce a calibration curve at 285 nm. The concentration of HMF present in each sample was calculated from the area under the curve (AUC) of HMF standards and the respective honey samples.

### 2.5 Biochemical characterization

#### 2.5.1 LC-MS analysis

The honey samples were extracted using water as a solvent to perform an extensive polyphenol profile of them. After that, a SpeedVac evaporator was used to concentrate the honey extracts ten times. The SPE-bound material and honey extracts were reconstituted in 5% v/v acetonitrile with 0.1% v/v formic acid. The resultant samples were analyzed using LCQ-DECA system, which included a binary gradient pump, photo diode array detector (PDAD), 3D ion trap mass analyzer, and mass spectrometer (Thermo Fisher Scientific, USA). A gradient flow was used which included solvent A (ultra-pure water with 0.1% (v/v) formic acid) and solvent B (acetonitrile with 0.1% (v/v) formic acid). Next, these gradients were used to elute 20 μL of samples with *t* = 0-5 min and 2% solvent B, as well as *t* = 5-35 min and 2-40% solvent B. The PDAD was used to scan 3 distinct channels (280, 365, and 520 nm) at a flow rate of 200 μL/min using a C18 column (Synergi HydroC18 with polar end capping, 2 mm × 150 mm, Phenomenex Ltd., UK). Compound identities were confirmed by MS/MS spectral matching against the NIST 2020 library (match factor > 85%) for all components, with key bioactives doubly validated. SRplot was used to plot the principal component analysis (PCA) of individual components for both polyphenols and terpenoids found in these four honey samples.

#### 2.5.2 NMR analysis

For ^1^H NMR spectroscopy, honey samples (100 mg) were dissolved in 1 mL of 99.9% deuterated water (D_2_O, Sigma-Aldrich, USA) and vortexed until completely dissolved. Solutions were then transferred to 5 mm NMR tubes (Wilmad LabGlass, USA) and analyzed using a 400 MHz Bruker Avance III spectrometer equipped with a 5 mm BBI probe at 25 °C. Spectral acquisition parameters included 64 scans, 8.2 μs 90° pulse, 11 ppm spectral width, 4.0 s relaxation delay, and 32 K time domain points. Water suppression was achieved using presaturation during the relaxation delay. Fourier transformation, phase correction, baseline correction, and chemical shift calibration (TMS at 0.0 ppm) were performed using TopSpin 4.0 software (Bruker, USA). Spectral regions (0.5-9.5 ppm) were integrated to quantify carbohydrate (3.2-5.5 ppm), phenolic (6.5-8.5 ppm), and amino acid (0.8-3.0 ppm) signals. Fingerprint regions were compared across samples (CiH, CoH, LyH, MuH) to identify cultivar-specific signatures; spectra were normalized to total integral area for quantitative comparison and performed in triplicate.

#### 2.5.3 FTIR analysis

Fourier Transform Infrared (FTIR) spectroscopy was conducted using a Nicolet iS50 FTIR spectrometer (Thermo Fisher Scientific, USA) equipped with a diamond Attenuated Total Reflectance (ATR) crystal. Honey samples (10 μL) were directly applied to the ATR crystal surface, forming a thin uniform film, and dried at ambient conditions for 5 min to minimize water interference. Spectra were recorded from 4000 to 500 cm^−1^ at 4 cm^−1^ resolution with 128 scans per sample using OMNIC Spectra software. Background spectra (clean ATR crystal) were subtracted automatically. Spectral preprocessing included apodization (Happ-Genzel), zero-filling (2 levels), and automatic baseline correction. Key spectral regions were analyzed: O-H/N-H stretching (3200-3600 cm^−1^), C-H stretching (2800-3000 cm^−1^), amide I/II bands (1600-1700 cm^−1^), C=O stretching (1700-1750 cm^−1^), fingerprint region (900-1500 cm^−1^), and C-O/C-OH stretching (1000-1300 cm^−1^). PCA was performed on second-derivative spectra (Savitzky-Golay, 13 points) using OriginPro 2023 to discriminate honey cultivars based on vibrational signatures. Triplicate measurements were averaged; standard deviations were <5% across spectral regions.

### 2.6 Nutritional characterization

#### 2.6.1 Total sugars (reducing and non-reducing) content

Both reducing and non-reducing sugars were tested using the Fehling solution method (Lomiso et al., 2021). Briefly, 5 g of different honey samples were dissolved in 100 mL of distilled water and filtered through filter paper and placed into a 250 mL volumetric flask. Following pipetting and preparation of the solution into a conical flask, 10 mL of diluted hydrogen chloride (1 N) was added and the mixture was allowed to boil for 5 min. After cooling, phenolphthalein with 10% NaOH (aqueous solution; *w*/*v*) was added to neutralize and then the solution was used for titration against the Fehling solution. The titre volume was reported for further investigation.

$$\text{Reducing sugars}\;\left(\%\right)=\left[\text{Factor}\;\left(4.95\right)\times \text{Dilution}\;\left(250\right)\right]/\left[\text{Titre}\times \text{Weight of sample}\times 10\right]$$

$$\text{Total sugars}\;\left(\%\right)=\left[\text{Factor}\;\left(4.95\right)\times \text{Dilution}\;\left(250\right)\times 2.5\right]/\left[\text{Titre}\times \text{Weight of sample}\times 10\right]$$

#### 2.6.2 Proline content

Proline content of the honey samples was determined according to the previously reported protocol (Moniruzzaman et al., 2013). Briefly, 50 mL of water and 5 g of honey were added to a beaker which was further diluted by adding 100 mL of distilled water. After that, 0.5 mL of the sample was mixed with 1 mL of ninhydrin solution and 1 mL of formic acid and allowed to boil in a boiling water bath for 15 min. The sample mixture was then transferred to another water bath and incubated for 10 min at 70 °C. Roughly 5 mL of 2-propanol was added to the sample mixture and the tubes were quickly sealed and allowed to cool for 45 min. The absorbance was measured at 510 nm with a spectrophotometer (BMG LabTech, FLUOstar OMEGA, Germany).

#### 2.6.3 Total protein content

The protein content was quantified using the Lowry method (Alarjani & Mohammed, 2024). Briefly, 0.1 mL of the protein extract (50% w/v honey sample) was mixed with 1 mL of alkaline copper reagent (prepared by combining 2% sodium carbonate in 0.1 N NaOH with 0.5% copper sulfate and 1% potassium sodium tartrate) and incubated for 10 min at room temperature. Subsequently, 0.1 mL of Folin-Ciocalteu reagent (diluted 1:1 with distilled water) was added, and the mixture was incubated in the dark for 30 min. The absorbance of the resultant blue colour was measured at 750 nm using a bovine serum albumin (BSA) standard curve (10-100 μg/0.1 mL in 0.15 M NaCl) for calibration.

#### 2.6.4 Total polyphenol content

Total polyphenol content in honey samples was measured according to previously published protocol (Lawag et al., 2023). In order to determine the total polyphenol content, 10-fold diluted 2.5 mL of Folin-Ciocalteu reagent was mixed with 10 μL of the sample extract. To distinguish from the other reducing compounds like reducing sugars, 2.5 mL of 7% sodium carbonate was added after 5 min and the mixture was allowed to incubate for 1 h at room temperature (Singleton et al., 1999). After that, the absorbance of the reaction mixture was recorded at 760 nm using a spectrophotometer (BMG LabTech, FLUOstar OMEGA, Germany). The total polyphenol content was represented in milligram of gallic acid equivalents (GAE) per gram of the sample.

#### 2.6.5 Total flavonoid content

Total flavonoid content in honey samples was measured according to an earlier report (Sultana et al., 2024). The content of the total flavonoids was determined by the aluminium chloride colourimetric technique. Briefly, 1 mL of sample extract was added to 4 mL of distilled water in a test tube. Next, 0.3 mL of 5% sodium nitrite was added followed by an incubation of 5 min. After this, 0.3 mL of 10% aluminium chloride was added to the test tube containing the mixture. The volume of the mixture was adjusted to 10 mL with the addition of 2.4 mL of distilled water. This 10 mL reaction mixture was thoroughly mixed with the help of a vortex machine. The absorbance of the reaction mixture was measured at 510 nm using a spectrophotometer (BMG LabTech, FLUOstar OMEGA, Germany). The representation of the total flavonoid content was in milligram of rutin equivalents (RE) per gram of the sample.

#### 2.6.6 Total phenolic acid content

Total phenolic acid content in honey samples was measured according to a previously published protocol (Bertoncelj et al., 2007). Different honey samples were dissolved in warm distilled water at a concentration of 500 mg in 5 mL. The mixtures were then sonicated for 5 min to obtain clear solutions. From each solution, 100 μL (equivalent to 10 mg of fresh honey) was taken and added to 1 mL of Folin-Ciocalteu reagent that had been pre-diluted 1:10 with distilled water. The resulting mixture was vortexed for 2 min. After incubation, the absorbance was measured at 750 nm using a spectrophotometer (BMG LabTech, FLUOstar OMEGA, Germany). The representation of the total phenolic acids was in milligram GAE per gram of the sample.

#### 2.6.7 DPPH scavenging activity

DPPH scavenging activity of the honey samples was estimated based on an earlier published protocol (Chua et al., 2013). A sample extract of 1 mL was prepared in a test tube in which 2 mL of 1 mM methanolic DPPH was added. This solution was thoroughly mixed and incubated at 37 °C for 30 min. In the blank incubation, no sample extract was added. At 515 nm the change in the absorbance was measured and the inhibition percentage was calculated by the following formula:

$$\text{Inhibition}\;\left(\%\right)=\left[\left(A515\;\text{Control}-A515\;\text{Sample}\right)/A515\;\text{Control}\right]\times 100$$

#### 2.6.8 H_2_O_2_ scavenging activity

H_2_O_2_ scavenging activity of the honey samples was estimated according to a previously reported protocol (Mohammedi, 2021). Hydrogen peroxide solution of 40 mM concentration was prepared in a 50 mM phosphate buffer (pH 7.4) solution and the absorbance was measured at 230 nm. Then sample extract or standard of 1 mL volume was added to 2 mL of H_2_O_2_ solution. After 10 min of incubation, the absorbance was measured at 230 nm against blank. For blank, phosphate buffer was added without the addition of H_2_O_2_. The percentage of H_2_O_2_ scavenging activity was calculated by the formula:

$$H_{2}O_{2}\;\text{scavenge}\;\left(\%\right)=\left[\left(A230\;\text{Control}-A230\;\text{Sample}\right)∕A230\;\text{Control}\right]\times 100$$

### 2.7 Biological characterization for glucose-lowering activity

#### 2.7.1 α-Amylase inhibition assay

The α-amylase inhibition activity of different honey samples was carried out by the 2,3-dinitrosalicylic acid method as described earlier by Moein et al. (2022). The reaction system without the α-amylase was used as blank and acarbose as a positive control. The control condition used in this assay represented the negative control, where the reaction contained α-amylase but no honey sample or inhibitor, thereby exhibiting null inhibition (100% enzyme activity). The absorbance was measured at 540 nm using a microplate reader (BMG LabTech, FLUOstar OMEGA, Germany). The % inhibition of activity was calculated as

$$\left[\frac{X_{A}-X_{B}}{X_{A}}\right]*100$$

where, $X_{A}$= absorbance of control (100% enzyme activity), $X_{B}$= absorbance of samples.

#### 2.7.2 α-Glucosidase inhibition assay

The α-glucosidase inhibition activity of different honey samples was carried out by the 4-nitrophenyl α-D-glucopyranoside method as described by Moein et al., earlier (Moein et al., 2022). The reaction system without α-glucosidase was used as blank and acarbose was used as a positive control. Similar to the α-amylase assay, the control condition served as the negative control, containing α-glucosidase but no honey sample or inhibitor, and showed null inhibition (100% enzyme activity). The absorbance was measured at 405 nm using a microplate reader (BMG LabTech, FLUOstar OMEGA, Germany). The % inhibition of activity was calculated as

$$\left[\frac{X_{A}-X_{B}}{X_{A}}\right]*100$$

where, $X_{A}$= absorbance of control (100% enzyme activity), $X_{B}$= absorbance of samples.

#### 2.7.3 Cell culture and treatments

HepG2 cells, a human hepatoma cell line, were obtained from the National Centre for Cell Sciences (NCCS), Pune, India. The cells were grown at 37 °C in a 95% humidified CO_2_ incubator in DMEM supplemented with 1.1% antibiotic-antimycotic cocktail (100 U/mL penicillin, 100 μg/mL streptomycin, and 0.25 μg/mL amphotericin B; Himedia, Mumbai, India) and 10% FBS. The HepG2 cells were treated with 0.5 mM palmitic acid-containing incomplete media for 24 h to induce hepatic steatosis (Ghosh et al., 2025, Ghosh et al., 2026). Then, different honey samples (CiH, CoH, LyH and MuH) were added in a dose-dependent manner, followed by 100 nM insulin to assess the glucose-lowering effect of these honey samples. Metformin (2 mM) was used as a positive control where applicable.

#### 2.7.4 Data normalization and re-expression

To address compositional variability, bioactivity data were normalized to total sugar content (*S*, g per 100 g honey) and total phenolic content (*P*, mg GAE per g honey), as quantified in Table 4. Similar approaches for normalizing bioactivity data based on total phenolic or sugar content have been reported in earlier studies of enzyme inhibition (Hogan et al., 2010; Strelow et al., 2012; Xiong et al., 2020). These precedents support the current normalization strategy to account for compositional variability across honey samples. Two types of normalization were applied, depending on the nature of the endpoint:

For inhibitory endpoints (IC₅₀ of α-amylase, α-glucosidase and the intracellular lipid accumulation), raw IC₅₀ was expressed as mg honey/mL. To obtain the concentration of sugars or phenolics present at the IC₅₀, the raw value was multiplied by the respective content.

*S* = total sugars (g per 100 g honey)

*P* = total phenolics (mg GAE per g honey)

Then:

$$\text{Sugar}-\text{normalized}\;{\mathrm{IC}}_{50}\;\left(\mathrm{mg}\;\text{sugar}/\mathrm{mL}\right)={\mathrm{IC}}^{50}\left(\mathrm{mg}\;\text{honey}/\mathrm{mL}\right)\times \frac{S}{100}$$

$$\text{Phenolic}-\text{normalized}\;{\mathrm{IC}}_{50}\;\left(\mathrm{mg}\;\mathrm{GAE}/\mathrm{mL}\right)=\mathrm{IC}₅₀\left(\mathrm{mg}\;\text{honey}/\mathrm{mL}\right)\times \frac{P}{1000}$$

For stimulatory/ameliorative endpoints (glucose uptake % and intracellular glycogen %), raw values are divided by the sugar or phenolic content to express the biological response per unit of that component.

*S* = total sugars (g per 100 g honey)

*P* = total phenolics (mg GAE per g honey)

Then:

$$\text{Sugar}-\text{normalized response}=\frac{\%\text{of response}}{S/100}$$

$$\text{Phenolic}-\text{normalized response}=\frac{\%\text{of response}}{\frac{P}{1000}}$$

These normalized values were calculated for α-amylase and α-glucosidase inhibition assays, glucose uptake, intracellular glycogen content, and lipid accumulation endpoints, but not for the MTT cytotoxicity assay due to known interferences from honey reducing sugars and phenolics with MTT reduction.

#### 2.7.5 Cytotoxicity assay

MTT assay was performed to study the cytotoxic effects of these honey samples based on the protocol reported earlier from our laboratory (Ghosh et al., 2025, Ghosh et al., 2026)**.** Briefly, 5 × 10^3^ HepG2 cells were seeded per well in a 96-well plate and incubated for 24 h. Next, the cells were treated with different concentrations of honey samples (20-100 mg/mL) for 24 h. Following the stipulated incubation period, 20 μL of 5 mg/mL MTT was added, and the mixture was incubated for 4 h at 37 °C. Subsequently, the MTT-containing media were replaced with 150 μL of DMSO to facilitate the dissolution of the formazan crystals. The absorbance was then measured at 570 nm using a microplate reader (BMG LabTech, FLUOstar OMEGA, Germany). The % of survival was calculated from the resultant readings as:

$$\left[\frac{A570_{\text{sample}}}{A570_{\text{control}}}\right]*100$$

#### 2.7.6 Glucose uptake assay and measurement of intracellular glycogen

Following the required treatments, glucose uptake was measured by taking 10 μL of conditioned media from each plate and assaying with a glucose estimation kit (Erba Mannheim, Germany) according to the manufacturer’s instructions. The glucose concentrations from the control and different treatment groups were subtracted from the blank (with no cells, only media) to obtain the amount of consumed intracellular glucose.

Intracellular glycogen was measured by the anthrone method as described earlier (Carroll et al., 1956). Briefly, the cells were homogenized with trichloroacetic acid and then centrifuged to discard the pelleted materials. Then, 5 volumes of 95% ethanol were added to the supernatant and incubated overnight at room temperature. The precipitated glycogen was dissolved in distilled water and then 5 volumes of anthrone reagent were added and mixed vigorously, followed by incubating in a water bath for 15 min at 95 °C. Finally, after cooling, the absorbance was taken at 620 nm in a spectrophotometer (BMG LabTech, FLUOstar OMEGA, Germany). Distilled water and anthrone reagents were mixed to make a solution to be used as blank.

#### 2.7.7 BODIPY 493/503 staining for intracellular lipid accumulation

BODIPY staining assay for identifying lipid droplets was performed according to previously published protocol (Varshney et al., 2018). Briefly, after the required incubations with palmitic acid to induce lipid accumulation intracellularly, the cells were treated with different honey samples (40 mg/mL) for 24 h. This was followed by washing the cells with PBS and fixed with 4% paraformaldehyde for 30 min at room temperature. Then the cells were incubated with 5 μM BODIPY 493/503 staining solution (dissolved in PBS) for 15 min. After that the cells were washed with PBS and the images were captured in a fluorescence microscope (EVOS FLoid, Invitrogen, Thermo Fisher, USA).

#### 2.7.8 Intracellular fatty acid quantification by GC-MS

Intracellular fatty acid content in HepG2 cells was quantified using gas chromatography-mass spectrometry (GC-MS) with flame ionization detection (FID; Agilent 8890, Agilent Technologies, CA, USA), adapted from established protocols for lipid profiling in insulin-resistant hepatocytes (Ghosh et al., 2025). Following treatments, the cells were washed twice with ice-cold PBS, scraped in 500 μL methanol:chloroform (2:1 *v*/v) containing 0.01% butylated hydroxytoluene (BHT) as antioxidant, and lipids extracted by the Folch method with chloroform:methanol (2:1 v/v) and 0.9% NaCl. The organic phase was evaporated under nitrogen, and total lipids (∼5 mg) were transesterified to fatty acid methyl esters (FAMEs) using 2 mL 14% BF₃-methanol at 100 °C for 90 min. FAMEs were extracted twice with hexane (2 mL), dried under nitrogen, and resuspended in hexane (1 mL). GC-MS analysis used a DB-23 column (60 m × 0.25 mm i.d., 0.25 μm film; Agilent J&W), with helium carrier gas at 1.2 mL/min. Oven temperature program was: 140 °C hold 2 min, ramp 5 °C/min to 220 °C hold 10 min. Injector 250 °C, FID detector 260 °C. FAMEs were identified by comparison to Supelco 37 Component FAME Mix standard (Sigma-Aldrich, retention times ±0.05 min) and NIST 2020 library matching (>90% similarity), quantified via peak area normalization to internal standard (tricosanoic acid C23:0, 50 μg/mL). Fatty acids were categorized as saturated (SFA: C14:0, C16:0, C18:0), monounsaturated (MUFA: C16:1n-7, C18:1n-9), and polyunsaturated (PUFA: C18:2n-6, C20:4n-6, C18:3n-3). Data expressed as % total fatty acids. Total SFA, MUFA, PUFA levels and ratios (e.g., SFA/PUFA) were calculated to assess treatment-induced desaturation shifts.

#### 2.7.9 Immunoblot analysis

HepG2 cells were seeded at 1 × 10^6^ cells per well in 6-well plates and incubated overnight for 80-90% confluency. Following treatments (0.5 mM palmitic acid for 24 h to induce insulin resistance, followed by honey samples at 40 mg/mL and/or 100 nM insulin for 1 h), cells were washed twice with ice-cold PBS and lysed in RIPA buffer (50 mM Tris-HCl pH 7.4, 150 mM NaCl, 1% NP-40, 0.5% sodium deoxycholate, 0.1% SDS) supplemented with protease/phosphatase inhibitor cocktail (1:100, Sigma-Aldrich) and 1 mM PMSF on ice for 30 min with intermittent vortexing. The lysates were then centrifuged at 13,000 ×*g* for 20 min at 4 °C, and supernatants (whole-cell lysates) were collected; protein concentration was determined using Bradford method with BSA as standards. Equal protein (50 μg) was resolved on 10% SDS-PAGE gels, transferred to PVDF membranes (0.45 μm, Millipore, USA) in transfer buffer (25 mM Tris, 192 mM glycine, 20% methanol), and blocked in 3% BSA/TBST (TBS + 0.1% Tween-20). Membranes were probed overnight at 4 °C with primary antibodies, and anti-β-actin as a loading control. After washing 3-times with TBST (10 min each), membranes were incubated with HRP-conjugated secondary antibodies and developed using the enhanced chemiluminescence (ECL) method with Luminol reagents (Giri Diagnostic & Kits & Reagent Private Limited, Dehradun, India), and images were captured using ChemiDoc XRS+ (BioRad, USA). Band intensities were quantified by ImageJ (NIH), normalized to β-actin, and expressed as fold-change relative to control. Experiments were performed in three independent biological replicates.

### 2.8 Principal component analysis (PCA)

To investigate the underlying structure and relationships among the four honey cultivars based on their comprehensive physicochemical and bioactive properties, PCA was employed using Python (Python Software Foundation, Wilmington, DE, USA). The analysis was performed using the PCA module from the Scikit-Learn library (v1.0.2) (Pedregosa et al., 2011). Data handling and structuring were done using the Pandas library (v1.3.5) (McKinney, 2010). Prior to analysis, the data matrix was mean-centered and auto-scaled (unit variance scaling) using the StandardScaler function from Scikit-Learn to ensure that all variables contributed equally regardless of their original measurement scales. The results were visualized using Matplotlib (v3.5.1) (Hunter, 2007) to generate score plots and loading plots. The score plot was used to visualize sample groupings, while the loading plot identified the original variables most influential in defining the observed clusters. A biplot was also generated to simultaneously display both the sample scores and variable loadings, facilitating the interpretation of relationships between the honey samples and their measured properties.

### 2.9 Statistical analysis

All the experiments have been performed in three independent biological replicates. The statistical analysis was performed using GraphPad Prism software (version 5). One-way ANOVA (Tukey's post hoc test) was used to analyze more than two datasets, and two-way ANOVA with Bonferroni's post hoc test was used to compare more than one parameter for the same datasets. Multiple comparison corrections were applied using the Benjamini-Hochberg false discovery rate (FDR) procedure to control for family-wise error rates across all post-hoc comparisons. Effect sizes were calculated as Cohen's d for pairwise comparisons and partial eta-squared (η^2^_p) for ANOVA analyses, with values reported alongside 95% confidence intervals (CIs). Throughout the analysis, a 95% confidence level was used, and a statistical likelihood of equal to or less than 0.05 (*p*-value) was considered significant. A parametric test (e.g., ANOVA) was applied after verifying the assumptions of normality and homoscedasticity. Normality was assessed using the Shapiro-Wilk test. Homoscedasticity (equality of variances) was evaluated using Levene’s test for ANOVA where appropriate. The statistical significances for *p* < 0.05, *p* < 0.01, and *p* < 0.001 probabilities were indicated by the symbols ‘^a^’, ‘^aa^’, and ‘^aaa^’, respectively, in the figures, wherever applicable.

## 3 Results

### 3.1 Pollen analysis by SEM

The honey samples collected from various locations in India (Fig. 1A) were subjected to SEM analysis to examine their floral sources. SEM analysis revealed distinct pollen morphologies corresponding to the dominant botanical origins of the four honey samples. In CiH (Fig. 1Ba), most pollen grains belonged to the Lauraceae family, exhibiting elliptical shapes with a reticulate exine pattern and a single sulcus aperture. CoH (Fig. 1Bb) was predominantly characterized by Arecaceae pollen, displaying monocolpate, spherical but ellipsoidal shape with a prominent furrow and grains with a finely granulated surface. LyH (Fig. 1Bc) contained a high frequency of Sapindaceae pollen, identified by their tricolporate structure and striate exine ornamentation. Meanwhile, MuH (Fig. 1Bd) primarily consisted of Brassicaceae pollen, showing small, prolate grains with a distinct tricolpate configuration and finely reticulated surface. Despite differences in taxonomic origin, all pollen types shared architectural similarities, including well-defined apertures and sculptured exine patterns, which are typical adaptations for entomophilous pollination. The morphological consistency within each honey sample confirmed their unifloral nature, as further supported by comparative palynological references. Supplementary table, Table S1, summerizes detailed melissopalynological analysis of pollen grains for these honey samples.

### 3.2 Biophysical characterization

**Fig. 2:**
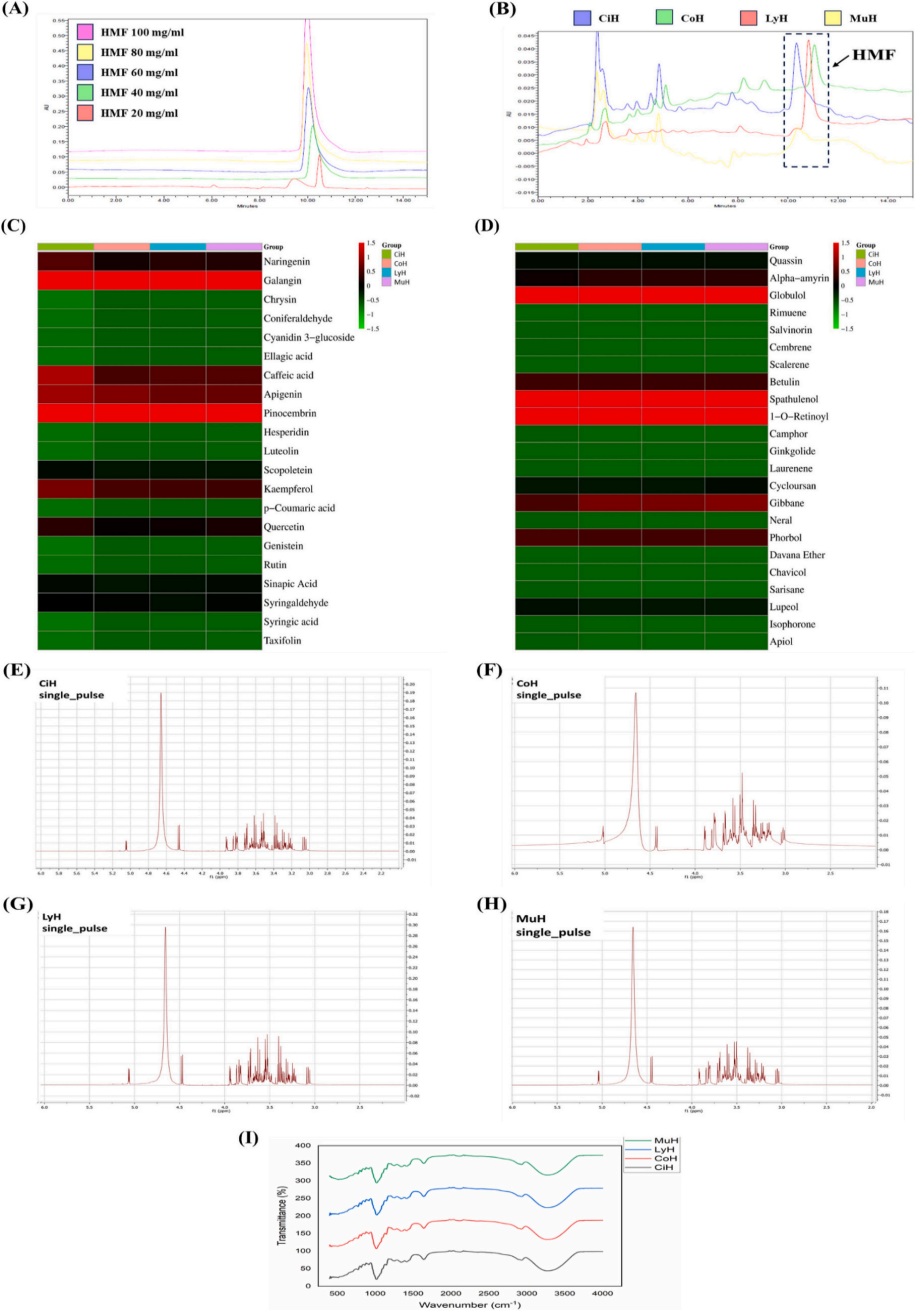
Phytochemical compositional analysis of different Indian honeys by HPLC and LC-MS. Representative chromatogram of HPLC analysis of HMF content with increasing concentrations of [A] HMF standard and [B] different honey samples (CiH, CoH, LyH and MuH). Representative heatmap indicating the comparative bioactive compounds [C] polyphenols and [D] terpenoids in different honeys. [E-H] Representative chromatograms of ^1^H NMR analysis of different Indian honey samples. [I] Representative FTIR profiling of different Indian honey samples. HMF, hydroxymethylfurfural; CiH, cinnamon honey; CoH, coconut honey; LyH, lychee honey; MuH, mustard honey; FTIR, Fourier Transform Infrared Spectroscopy; NMR, Nuclear Magnetic Resonance.

Table 1 displays the biophysical characterization of different Indian monofloral honey samples, which includes parameters such as pH, electrical conductivity, ash content, moisture content, colour, and the HMF content. The pH of honey samples was in the range between 3.58 and 3.83. All samples showed similar acidic pH which follows standards set by the European Union (EU) and the international Codex (3.6-4.3) (Bogdanov et al., 1999; Gupta et al., 2014). Out of the four samples, MuH had the most acidic pH (3.54 ± 0.02), resembling its various natural acids and enzymes enrichment. Electrical conductivity and ash content of honey (for example, 0.4-0.55 mS/cm for manuka honey (Luca et al., 2025; Moniruzzaman et al., 2013; Nguyen et al., 2018)) should be below 0.8 mS/cm and 0.6%, respectively as permitted by EU and International Codex (Bogdanov et al., 1999; Gupta et al., 2014). However, some exceptions (like chestnut and linden honey) are present, with higher electrical conductivity (Akgün et al., 2021; Barbarić et al., 2025). The electrical conductivity values for chestnut honey (0.56-1.12 mS/cm) (Saral, 2023) and linden honey (0.8-1.2 mS/cm) (Barbarić et al., 2025) exceed typical floral honey limits (<0.8 mS/cm), likely due to their higher mineral content (e.g., sodium, potassium, calcium) and phenolic compounds derived from the dark, nutrient-rich nectar of chestnut flowers and the bioactive constituents of linden blossoms (Kędzierska-Matysek et al., 2018; Küçük et al., 2007). The electrical conductivity and ash content of CoH, LyH, and MuH were within the limit, but for CiH, both the parameters were above the permissible range, having 0.91 ± 0.003 mS/cm and 0.67 ± 0.003% for electrical conductivity and ash content, respectively. This indicated the presence of a higher level of carbon and other minerals in CiH compared to the other three honey samples. Honeys with mineral content and electrical conductivity higher than the permissible range are often considered dark honey or honeydew honey (honey with a deep, rich colour and a more intense, robust flavour profile generally obtained from the nectar source). Moisture content directly impacts honey's shelf life. Honey with higher moisture content (above 20%) is more likely to ferment, especially if stored improperly. Moisture content of honey should be less than 20% as set by the EU and International Codex; otherwise, it will be susceptible to yeast contamination (Bogdanov et al., 1999; Gupta et al., 2014). The moisture content in all the honey samples was found to be within the limit ranging from 11.66 ± 1.08 to 16.33 ± 0.04%. Among all the honey samples tested, MuH and LyH had the least (11.66 ± 1.08%) and highest (16.33 ± 0.04%) amount of moisture content, respectively. The honey colour is affected by different parameters, including the presence of pollen, HMF, etc. (Cabrera & Santander, 2022; Sakač et al., 2019). The colour of honey is a key indicator of its composition and quality, as it is influenced by factors such as the type of flowers the bees foraged on, the presence of certain compounds, and its processing methods (Sun et al., 2025). Darker honey typically contains more antioxidants, vitamins, and minerals such as iron, potassium, and magnesium, compared to lighter honey (Barreiros et al., 2024). According to the Pfund scale, LyH was light amber in colour, MuH was amber in colour while CoH and CiH were dark amber in colour, depicting the richness of natural compounds (Bodor et al., 2021). Additionally, it is to be noted that honey darkens over time as it ages due to oxidation and the Maillard reaction (interaction between sugars and amino acids), providing additional information about the age of honey if it differs from the expected colour (Seraglio et al., 2019). HMF concentration serves as a well-established indicator of honey freshness. Fresh honey typically contains minimal or no HMF, and its presence increases over time due to processing or aging. This rise in HMF concentration is a reliable measure of honey quality. HMF content of all the honey was well below the limit of 40 mg/kg as recommended by the EU and International Codex (Bogdanov et al., 1999; Gupta et al., 2014). Out of all samples, MuH and LyH were observed to have the lowest and highest amounts of HMF (11.62 ± 1.1 and 13.86 ± 1.03 mg/kg), respectively **(**Table 1 and Fig. 2A and 2B**)**.

**Table 1:** Biophysical characterization of different Indian honey samples.

Parameters	Honey samples			
	CiH	CoH	LyH	MuH
pH	3.58 ± 0.004	3.79 ± 0.02^aaa^	3.83 ± 0.01^aaa,ns^	3.54 ± 0.02^ns,bbb,ccc^
Electrical conductivity (mS/cm)	0.91 ± 0.003	0.81 ± 0.003^aaa^	0.41 ± 0.004^aaa,bbb^	0.67 ± 0.003^aaa,bbb,ccc^
Ash content (%)	0.67 ± 0.003	0.58 ± 0.003^aaa^	0.25 ± 0.003^aaa,bbb^	0.46 ± 0.002^aaa,bbb,ccc^
Moisture content (%)	14.66 ± 0.4	13.33 ± 0.4^ns^	16.33 ± 0.4^ns,b^	11.66 ± 1.08^a,ns,cc^
Colour (mm)	138.28 ± 82	137.51 ± 0.54^ns^	66.87 ± 2^aaa,bbb^	110.78 ± 2.94^aaa,bbb,ccc^
HMF (mg/kg)	13.03 ± 1.2	11.91 ± 0.78^aa^	13.86 ± 1.03^ns,bb^	11.62 ± 1.1^aa,ns,bb^

Data are the Mean ± SEM of three independent experiments. ‘a’ denotes significant difference between CiH and CoH/LyH/MuH; ‘b’ denotes significant difference between CoH and LyH/MuH; ‘c’ denotes significant difference between LyH and MuH. Either one, two or three numbers of these symbols (‘a’, ‘b’ or ‘c’) were used to represent the level of significance i.e., *p* < 0.05, *p* < 0.01 and *p* < 0.001, respectively. Statistical analysis was by One-way ANOVA (η^2^_p = 0.92) followed by Tukey's HSD with Benjamini-Hochberg FDR correction (q < 0.05). Key effect sizes (Cohen's d) and 95% Cis are presented in the Supplementary Table, Table S8. ‘ns’, non-significant; CiH, cinnamon honey; CoH, coconut honey; LyH, lychee honey; MuH, mustard honey.

### 3.3 Biochemical characterization

Table 2 and Table 3 represent different biochemical compositions of various honey samples as tested in this study. Table 2 summarizes a total of twenty-two polyphenols identified in different honey samples. The concentration of each polyphenol among the four honey samples differed significantly. Pinocembrin was the predominant polyphenol in CoH, LyH and MuH (0.932, 1.551 and 1.417 μg/g, respectively), while in CiH, galangin (0.653 μg/g) was found in the highest amount, depicting the diversity of floral sources of these honeys. However, chrysin, in all the honey samples, was not as high as expected to be a major polyphenolic constituent of many honeys and it ranged between 0.00017 and 0.00038 μg/g. This could be attributed to geographical location and floral types foraged by the bees. In addition, caffeic acid, apigenin and kaempferol were the other predominant polyphenols present in most of the honey samples, albeit with marginally variable amounts among them (Table 2). Table 3 displays the terpenoid composition of different honey. A total of twenty-four different terpenoids were identified of varying concentrations in the honey samples as tested. Among them, 1-O-retinoyl was the predominant terpenoid found in all the honey samples ranging from 0.977 to 1.5 μg/g (CoH and MuH having the lowest and highest amount of 1-O-retinoyl, respectively). Likewise, rimuene was the terpenoid present in the least amount in all the honey samples, ranging from 0.00021 to 0.00098 μg/g. Among the four samples, CoH and LyH had the lowest and highest amount of rimuene, respectively. Out of the other terpenoids, quassin, globulol, betulin, spathulenol, cycloursan, gibbane, phorbol and lupeol were the predominant terpenoids present in almost all the honey samples tested with varying concentrations. Supplementary Tables, Table S2 and S3, provide the retention time for the individual polyphenols and terpenoids as shown in Table 2, Table 3, respectively. Fig. 2C and 2D show the comparative heatmap of different polyphenols and terpenoids, respectively, found in various honey samples. The polyphenol heatmap (Fig. 2C) shows a distinct clustering pattern, with LyH and MuH exhibiting higher intensities of key polyphenols, indicating a richer antioxidant profile compared to CiH and CoH. The terpenoid heatmap (Fig. 2D) displays clear differentiation where CiH and CoH are enriched in specific terpenoids, suggesting a unique phytochemical composition distinct from LyH and MuH. The MS/MS fragmentation patterns for the flavonoids and terpenoids of all the honey samples detected in LC-MS analysis are shown in the Supplementary Tables, Table S4 and Table S5. To contextualize the phytochemical richness of the Indian monofloral honeys, a sample of manuka honey (*Leptospermum scoparium*) was included as a benchmark. Manuka honey is globally renowned for its distinct therapeutic properties, which are linked to a unique set of bioactive compounds and a specific array of phenolic markers. This reference honey is well-documented to contain a characteristic suite of compounds which are largely absent in other honey types and serve as key indicators of its botanical origin (Oelschlaegel et al., 2012). As shown in Supplementary Tables, Table S6 (polyphenols) and S7 (sugars), a distinct polyphenolic profile for manuka honey was revealed. This profile is notably different from the Indian honeys, which are characterized by high concentrations of common flavonoids such as pinocembrin, galangin, and kaempferol. In contrast, the manuka sample exhibited the presence of its characteristic marker compounds, including pinobanksin, tricetin, acacetin and 2-Hydroxycinnamic acid, which were absent in all those Indian honey samples. The instrument-generated analysis reports for the polyphenols and terpenoid profiling are provided in the Supplementary Figures, Fig. S1-S8. For manuka honey, the analysis report for polyphenol and sugar profiling are provided in the Supplementary Figures, Fig. S9 and S10, respectively. Fig. 2E-H present high-resolution ^1^H NMR spectra (in D₂O), each dominated by anomeric α/β-glucose/glucose signals (δ 5.2-5.4 ppm, doublet patterns) and a dense envelope of sugar proton multiplets (δ 3.2-4.2 ppm) from fructose, glucose, and minor oligosaccharides, with baseline distortions minimized for clarity. CiH (Fig. 2E) shows the cleanest spectrum with prominent fructose H-1 at ∼4.0 ppm, CoH (Fig. 2F) reveals slightly elevated signals at δ 1.2-1.5 ppm hinting at trace amino acids or volatiles, LyH (Fig. 2G) displays minor upfield shifts in the anomeric region suggesting varietal monosaccharide ratios, and MuH (Fig. 2H) features broader sugar clusters indicative of diverse oligosaccharide impurities. Fig. 2I displays the FTIR absorbance spectra across 500-4000 cm^−1^, where all samples exhibited hallmark carbohydrate bands: a broad O-H stretching vibration (∼3300 cm^−1^) from hydrogen-bonded hydroxyls, sharp C-H stretches (∼2930 and 2850 cm^−1^) from alkyl chains, intense C=O stretching (∼1640 cm^−1^) indicative of amide or carbonyl moieties, and fingerprint region peaks (∼1100-1000 cm^−1^) from C-O-C glycosidic linkages and sugar ring modes. While peak positions align closely, MuH shows elevated intensities in the 1200-900 cm^−1^ region suggesting higher polysaccharide content. Simultaneously, LyH displayed a pronounced shoulder at ∼1740 cm^−1^ possibly from trace amount of esters. In the same assay, while CoH showed broader O-H bands implying greater water activity, CiH exhibited sharper C-H peaks consistent with higher lipid-like impurities or volatiles, collectively underscoring batch-specific functional group abundance amid a uniform honey matrix. These orthogonal FTIR and NMR datasets robustly affirm the samples' high purity and authenticity, as evidenced by the absence of adulterant markers (e.g., no sharp synthetic C≡N or ester peaks in FTIR, no exogenous alcohols or HMF spikes in NMR), strong congruence with natural honey literature signatures, and sufficient intra-sample consistency to enable reliable generalization despite variable floral origins.

**Table 2:** Polyphenol profiling of different Indian honey samples by LC-MS.

Polyphenols	CiH	CoH	LyH	MuH
	Concentration (μg/g)	Concentration (μg/g)	Concentration (μg/g)	Concentration (μg/g)
Fisetin	0.144	0.110	0.199	0.2
Naringenin	0.239	0.169	0.326	0.301
Galangin	0.653	0.495	0.890	0.820
Chrysin	0.00018	0.00017	0.00037	0.00038
Coniferaldehyde	0.002	0.002	0.004	0.004
Cyanidin 3-glucoside	0.006	0.004	0.008	0.008
Ellagic acid	0.001	0.001	0.002	0.002
Caffeic acid	0.343	0.246	0.453	0.417
Apigenin	0.326	0.325	0.510	0.464
Pinocembrin	0.479	0.932	1.551	1.417
Hesperidin	0.001	0.001	0.002	0.002
Luteolin	0.0024	0.002	0.003	0.003
Scopoletin	0.124	0.102	0.186	0.169
Kaempferol	0.279	0.247	0.411	0.375
p-Coumaric acid	0.005	0.004	0.007	0.007
Quercetin	0.190	0.149	0.272	0.278
Genistein	0.00035	0.00038	0.00074	0.00074
Rutin	0.001	0.001	0.002	0.002
Sinapic acid	0.121	0.105	0.192	0.195
Syringaldehyde	0.142	0.132	0.194	0.197
Syringic acid	0.00061	0.00083	0.001	0.001
Taxifolin	0.002	0.002	0.003	0.003

CiH, cinnamon honey; CoH, coconut honey; LyH, lychee honey; MuH, mustard honey.

**Table 3:** Terpenoid profiling of different Indian honey samples by LC-MS.

Terpenoids	CiH	CoH	LyH	MuH
	Concentration (μg/g)	Concentration (μg/g)	Concentration (μg/g)	Concentration (μg/g)
Bilobalide	0.001	0.001	0.002	0.002
Quassin	0.156	0.155	0.230	0.238
Alpha-amyrin	0.213	0.275	0.407	0.423
Globulol	0.801	0.795	1.176	1.221
Rimuene	0.00025	0.00021	0.00098	0.00089
Salvinorin	0.002	0.003	0.005	0.005
Cembrene	0.004	0.004	0.005	0.005
Scalerene	0.001	0.001	0.001	0.001
Betulin	0.315	0.313	0.463	0.481
Spathulenol	0.829	0.823	1.217	1.264
1-O-Retinoyl	0.984	0.977	1.445	1.5
Camphor	0.002	0.002	0.003	0.003
Ginkgolide	0.001	0.001	0.002	0.002
Laurenene	0.005	0.005	0.007	0.008
Cycloursan	0.145	0.144	0.213	0.253
Gibbane	0.325	0.420	0.621	0.676
Neral	0.002	0.003	0.004	0.005
Phorbol	0.329.	0.327	0.483	0.526
Davana Ether	0.001	0.001	0.002	0.002
Chavicol	0.001	0.001	0.002	0.002
Sarisane	0.002	0.002	0.003	0.004
Lupeol	0.146	0.145	0.214	0.235
Isophorone	0.002	0.002	0.004	0.004
Apiol	0.002	0.002	0.002	0.002

CiH, cinnamon honey; CoH, coconut honey; LyH, lychee honey; MuH, mustard honey.

### 3.4 Nutritional characterization

Table 4 shows the nutritional characterization of different Indian honeys, which includes parameters such as total sugar, reducing sugar, non-reducing sugar, total polyphenols, total its long shelf life. This natural preservation property makes honey resistant to spoilage. Additionally, reducing sugar content is a key parameter in determining honey quality. A value below 60% may indicate adulteration or poor-quality honey, while a higher percentage confirms its natural authenticity and proper composition. LyH and MuH had the highest percentage of reducing sugar (almost 76%), while CoH had the lowest percentage (almost 69%). All the honey samples had more than 60% reducing sugar content, which is the acceptable value as provided by the International Codex (Bogdanov et al., 1999; Gupta et al., 2014). On the other hand, the percentage of non-reducing sugar in CiH, CoH, LyH, and MuH was 2.36 ± 0.36, 5.42 ± 0.25, 3.19 ± 0.9 and 2.2 ± 1.6, respectively. Non-reducing sugar primarily includes sucrose, and its presence above 5% indicates possible adulteration with sucrose (Aljohar et al., 2018). Except for CoH, all the other honey samples had the permissible range of non-reducing sugar, indicative of their purity, while for CoH, there is a possibility of added sugar in it.

**Table 4:** Nutritional characterization of different Indian honey samples.

Parameters	Honey samples			
	CiH	CoH	LyH	MuH
Total sugar (%)	74.43 ± 1.2	77.56 ± 1.76^ns^	79.62 ± 1.86^a,b^	78.66 ± 0.55^a,b,ns^
Reducing sugar (%)	71.89 ± 0.98	69.2 ± 1.13^aa^	76.48 ± 1.32^aaa,bbb^	76.13 ± 0.43^aa,bbb,ccc^
Non-reducing sugar (%)	2.36 ± 0.36	5.42 ± 0.25^a^	3.19 ± 0.9^aaa,bbb^	2.2 ± 1.6^ns,b,ccc^
Total polyphenols (μg/g)	1168.53 ± 32.56	1086.94 ± 31.64^ns^	1435.66±42.58^ns,ns^	1453.98±23.34^ns,ns,ns^
Total flavonoids (μg/g)	296.41 ± 4.28	241.97 ± 2.33^aaa^	269.35 ± 2.39^a,bb^	211.92±4.79^aaa,bbb,ccc^
Total phenolic acids (μg/g)	682.37 ± 2.53	651.76 ± 3.8^a^	310.89 ± 8.77^aaa,bbb^	670.63±3.08^ns,ns,ccc^
Total protein content (mg/g)	0.61 ± 0.05	0.86 ± 0.19^a^	0.66 ± 0.15^ns,b^	0.64 ± 0.20^ns,b,ns^
Proline content (mg/g)	1.98 ± 0.21	3.38 ± 0.18^aaa^	1.89 ± 0.2^aaa,bbb^	2.94 ± 0.13^aaa,ns,ccc^
H_2_O_2_ scavenging activity (%)	12.94 ± 1.23	9.83 ± 0.49^aa^	16.45 ± 0.45^a,bbb^	13.11 ± 0.51^ns,bb,cc^
DPPH scavenging activity (%)	15.62 ± 0.45	5.26 ± 0.26^aaa^	29.14 ± 0.2^aaa,bbb^	24.18 ± 0.56^aaa,bb,ccc^

Data are the Means ± SEM of three independent experiments. ‘a’ denotes significant difference between CiH and CoH/LyH/MuH; ‘b’ denotes significant difference between CoH and LyH/MuH; ‘c’ denotes significant difference between LyH and MuH, for the respective parameters tested. Either one, two, or three numbers of these symbols (‘a’, ‘b’, or ‘c’) were used to represent the level of significance, i.e., *p* < 0.05, *p* < 0.01, and *p* < 0.001, respectively. Statistical analysis was by One-way ANOVA (η^2^_p = 0.92) followed by Tukey's HSD with Benjamini-Hochberg FDR correction (q < 0.05). Key effect sizes (Cohen's d) and 95% Cis are presented in the Supplementary Table, Table S9. ‘ns’, non-significant; CiH, cinnamon honey; CoH, coconut honey; LyH, lychee honey; MuH, mustard honey.

When tested for the polyphenol content, LyH and MuH showed higher polyphenol content (>1400 μg/g GAE compared to CiH and CoH, although statistically non-significant (*p* < 0.05). However, such high polyphenol content has been reported by other previous studies also (Margolis, 2008; Ouchemoukh et al., 2007). Phenolic acid is one of the major classes of polyphenol compounds present in honey. LyH had a significantly lower amount of total phenolic acid content (310.89 ± 8.77 μg/g GAE) while the other three honeys had >600 μg/g GAE total phenolic acid content (*p* < 0.001). Flavonoids are among the most significant plant metabolites present in honey and are known to be responsible for many of the health-beneficial effects of honey. Total flavonoid content was expressed as rutin equivalent (μg/g). Among all the samples, CiH had the highest flavonoid content (296.41 ± 4.28 μg/g; statistically significant compared to other honey samples) while MuH had the least (211.92 ± 4.79 μg/g; statistically significant compared to other honey samples). Together, the total flavonoid content in all the honey was found to be >200 μg/g rutin equivalent, which refers to higher nutritional value and is consistent with the findings of the previous study (Margolis, 2008).

Total protein content in honey refers to the concentration of small protein fragments or peptides present in it. These proteins contribute to honey's nutritional and therapeutic properties. They play a critical role in antimicrobial activity, enhancing honey's ability to inhibit bacterial growth and promote wound healing. Additionally, proteins in honey have antioxidant properties, which help in combating oxidative stress, thereby supporting overall health. Although there is no defined limit for peptide content in honey, based on previous studies, it is assumed that total protein content in honey typically ranges from 0.02 to 1.0% of its dry weight (Ibrahim et al., 2021; Kaakeh & Gadelhak, 2005). In the current study, all the honey samples showed almost similar protein content, ranging from 0.61 ± 0.05 to 0.86 ± 0.19 mg/g (no statistical difference between different honey samples at *p* < 0.005), depicting typically moderate peptide content in these honey samples, indicative of their superior quality. Proline content in honey is an indicator of quality, as adulterated honey tends to have less proline content (Damto et al., 2024). Proline levels are often used as a marker to determine honey’s naturalness and maturity. Bees add proline to honey during nectar processing, and its high concentration reflects proper ripening. LyH and CoH had the lowest and highest proline content of 1.89 ± 0.2 mg/g and 3.38 ± 0.18 mg/g, respectively. All the honey had proline content of more than 0.18 mg/g, which is recommended according to the European Union (EU) and International Codex (Bogdanov et al., 1999; Gupta et al., 2014), resembling their freshness and authenticity.

In addition to evaluating the phytochemical composition, the antioxidant potential of these honey varieties was also examined. Two different antioxidant assays were performed to determine the free radical scavenging activity of honey, namely, H_2_O_2_ and DPPH scavenging activities, respectively. The assays were optimized and calibrated by using Trolox (positive control), as shown in the Supplementary Figure, Fig. S11. All the honeys showed similar trends of inhibition for both the free radical scavenging activities, albeit at varying efficiencies. As shown in Table 4, CoH imparted the least scavenging activities (9.83 ± 0.49 and 5.26 ± 0.26%) while LyH showed the highest free radical elimination activity (16.45 ± 0.45 and 29.14 ± 0.2%) for H_2_O_2_ and DPPH scavenging activities, respectively, among the four tested honey samples. In summary, based on the current set of data (Table 4), it could be concluded that in concurrence with variable phytochemical compositions (Table 2, Table 3), the nutritional profile of the tested honey samples also showed differential ranges in terms of content or activities. However, all four honey samples as tested here are roughly free of adulteration and of superior quality.

### 3.5 Enzyme inhibitory activities of honey

#### 3.5.1 α-Amylase enzyme inhibition

**Fig. 3:**
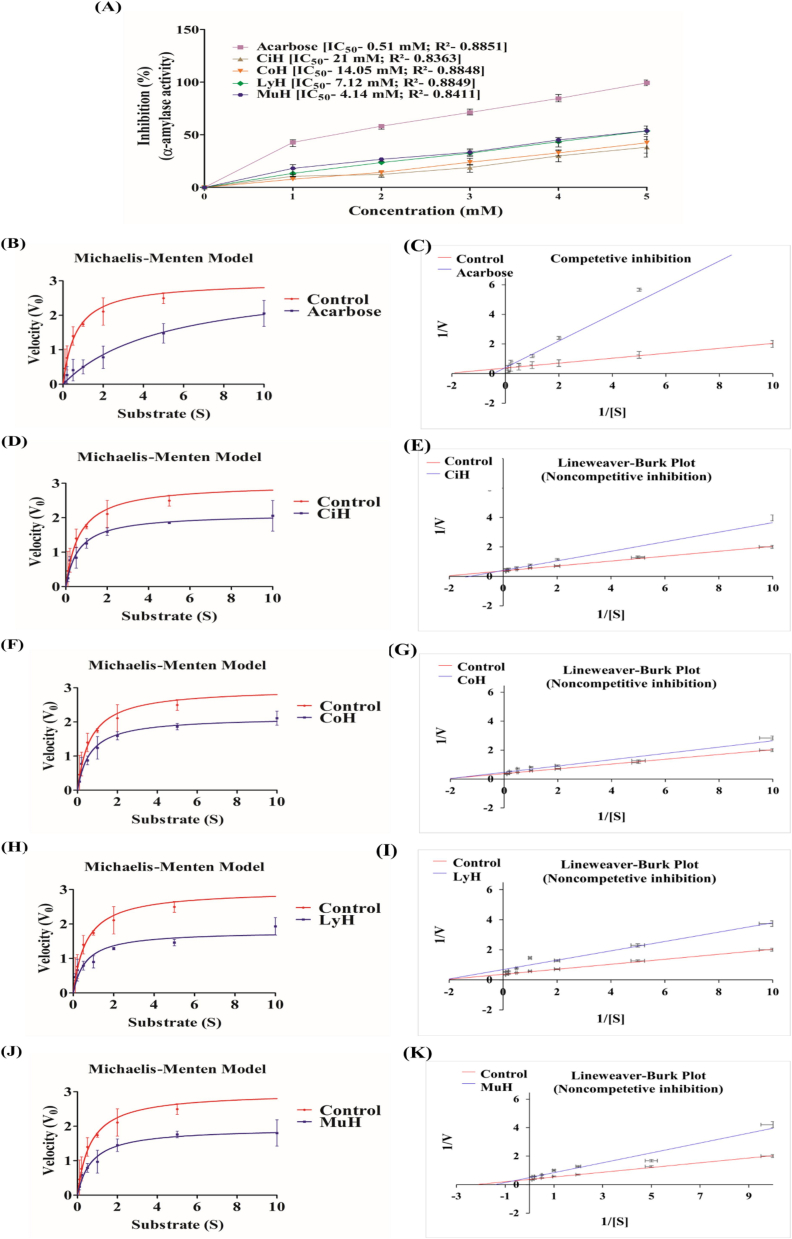
Inhibition of α-amylase activity by different Indian honeys. [A] Qualitative representation of α-amylase inhibition by acarbose and different honey samples with their IC_50_ values. Enzyme kinetic parameters for α-amylase inhibition as determined by the Michaelis-Menten graph for different honeys: [B] Acarbose, [D] CiH, [F] CoH, [H] LyH, and [J] MuH. Pattern of inhibition of α-amylase as determined by the Lineweaver-Burk plot for different honeys: [C] Acarbose, [E] CiH, [G] CoH, [I] LyH and [K] MuH. Data are Mean ± SEM of three independent biological experiments. CiH, cinnamon honey; CoH, coconut honey; LyH, lychee honey; MuH, mustard honey; V, velocity.

A preliminary evaluation of anti-diabetic or glucose-lowering activity was carried out by performing α-amylase inhibition assay against the honey samples and acarbose as positive control. α-Amylase is an enzyme that breaks down complex carbohydrates (starch) into simpler sugars, such as maltose and glucose, which are then absorbed into the bloodstream. By inhibiting this enzyme, the release of glucose into the bloodstream can be moderated, contributing to better glycaemic control. Results are displayed in Fig. 3A as the 50% inhibitory concentration (IC_50_) for α-amylase by the respective honey samples. Out of all the samples, MuH showed the highest inhibitory effect with lowest IC_50_ value of 4.14 mM (assuming the molecular weight of pure honey is 202 g/mol (Anidiobu, 2023)), followed by LyH (7.12 mM), CoH (14.05 mM) and CiH (21 mM), while acarbose exhibited an IC_50_ of 0.51 mM. This data indicates that, among all the honeys, MuH is the most potent for α-amylase inhibition with the highest inhibitory activity, and CiH is the least potent honey with the lowest inhibitory efficacy among these four tested honey samples. The inhibition kinetics parameters for α-amylase were also analyzed by calculating the K_m_ and V_max_ values from the Michaelis-Menten graph (Fig. 3B, D, F, H and J) and the type of inhibition was determined from the Lineweaver-Burk plot (Fig. 3C, E, G, I and K) for all the samples and are summarized in Table 5. All the honey samples showed non-competitive inhibition for α-amylase, based on enzyme kinetic assays demonstrating reduced maximum reaction velocity (V_max_) without significant changes to the Michaelis constant (K_m_), indicating inhibitor binding to the enzyme-substrate complex or allosteric sites rather than the active site, a hallmark of non-competitive inhibition, while acarbose has previously been reported to exhibit competitive inhibition (Ahmed et al., 2020). All the kinetic parameters are reported as untransformed raw measurements and in mM/min for V_max_ and mM for K_m_, unless otherwise stated.

**Table 5:** Enzyme kinetics parameters for α-amylase and α-glucosidase enzyme inhibition by different Indian honey samples.

Sample name	α-amylase inhibition				α-glucosidase inhibition			
	IC_50_(mM)	V_max_ (mM/min)	K_m_ (mM)	Type of inhibition	IC_50_ (mM)	V_max_ (mM/min)	K_m_ (mM)	Type of inhibition
Control (No inhibitor)	-	8.79	1.88	-	-	10.76	1.7	-
CiH	21	6.68	1.81	Non-Competitive	17.35	7.97	1.77	Non-Competitive
CoH	14.05	6.64	1.91	Non-Competitive	32.01	6.40	1.69	Non-Competitive
LyH	7.12	5.21	1.83	Non-Competitive	6.57	6.45	6.32	Mixed
MuH	4.14	4.85	1.96	Non-Competitive	5.95	6.80	1.69	Non-Competitive

#### 3.5.2 α-Glucosidase enzyme inhibition

**Fig. 4:**
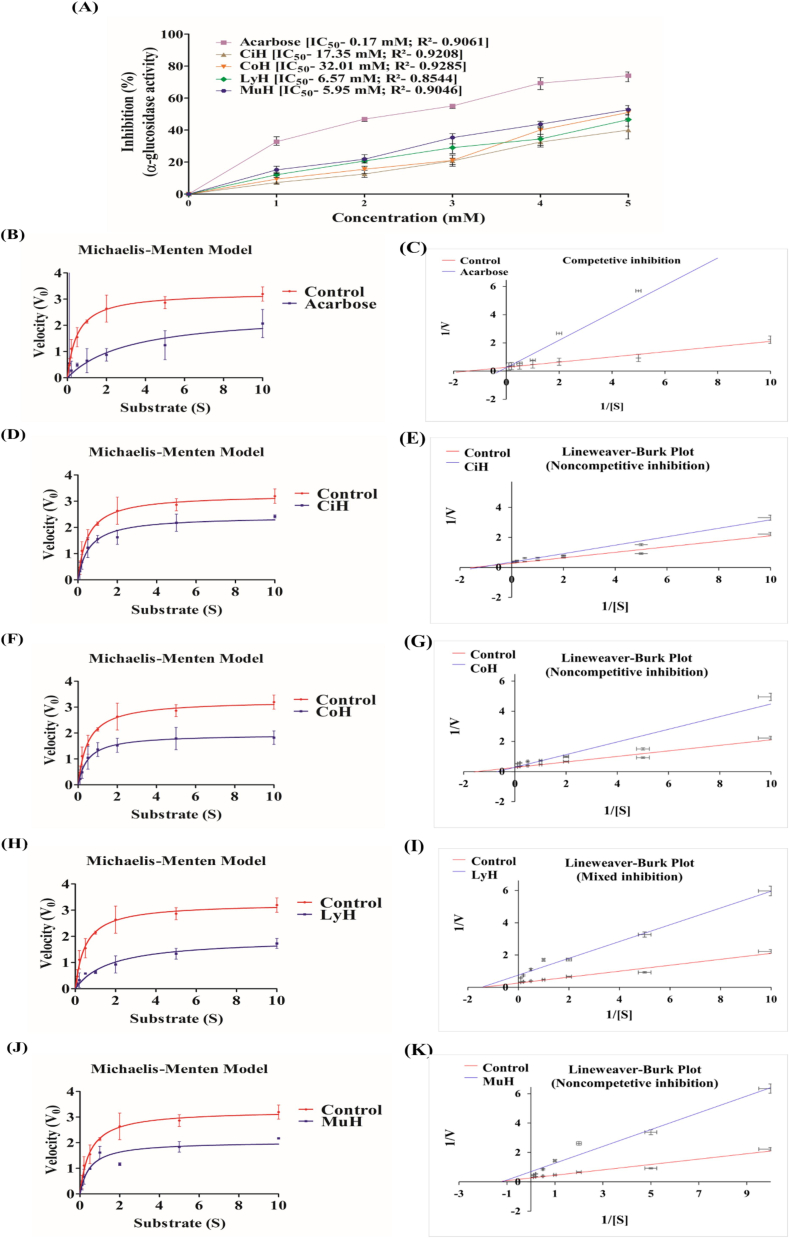
Inhibition of α-glucosidase activity by different Indian honeys. [A] Qualitative representation of α-glucosidase inhibition by acarbose and different honey samples with their IC_50_ values. Enzyme kinetic parameters for α-glucosidase inhibition as determined by the Michaelis-Menten graph for different honeys: [B] Acarbose, [D] CiH, [F] CoH, [H] LyH, and [J] MuH. Pattern of inhibition of α-glucosidase as determined by plotting the Lineweaver-Burk plot for different honeys: [C] Acarbose, [E] CiH, [G] CoH, [I] LyH and [K] MuH. Data are Mean ± SEM of three independent biological experiments. CiH, cinnamon honey; CoH, coconut honey; LyH, lychee honey; MuH, mustard honey.

Like α-amylase, the honey samples were found to be effective in inhibiting α-glucosidase activity as well *in vitro* **(**Fig. 4A**)**. α-Glucosidase is a key enzyme involved in the breakdown of complex carbohydrates into glucose in the small intestine. By inhibiting this enzyme, the release of glucose into the bloodstream is slowed down, which helps regulate postprandial (after-meal) blood glucose levels. Among all the honeys, MuH and LyH showed the highest inhibitory activity, as depicted by the lowest IC_50_ values of 5.95 and 6.57 mM, respectively, and thus are the most potent honeys among these four samples for the inhibition of α-glucosidase activity. CoH, with the lowest inhibitory effect (having an IC_50_ value of 32.01 mM), is the least potent honey toward the α-glucosidase inhibition, while CiH showed moderate α-glucosidase inhibition with an IC_50_ of 17.35 mM. The kinetic parameters, such as K_m_ and V_max_, were analyzed by following the Michaelis-Menten graph (Fig. 4B, D, F, H and J). According to the K_m_ and V_max_ values for all the honey samples and by following the Lineweaver-Burk plot, the type of inhibition of these honey samples was determined.

It was found that all the honey except LyH showed noncompetitive inhibition, while LyH exhibited mixed inhibition (Fig. 4C, E, G, I and K). LyH exhibited mixed inhibition because it uniquely showed both decreased V_max_ (reduced catalytic efficiency) and increased K_m_ (lower substrate affinity), unlike other honeys that followed a consistent pattern of non-competitive inhibition. This dual effect suggests LyH's polyphenols/terpenoids interact with both the enzyme's active site and allosteric regions, altering its kinetics differently. The IC_50_, K_m_ and V_max_ values for all the honey samples are summarized in Table 5. All the kinetic parameters are reported as untransformed raw measurements and in mM/min for V_max_ and mM for K_m_, unless otherwise stated.

To account for compositional differences influencing raw IC__50__ values, enzyme inhibitory potencies were normalized for total sugar content (g/g honey, Table 4) and total phenolic content (mg GAE/g honey, Table 4), converting IC_50_ from mM to mg honey/mL using average honey molecular weight of 202 g/mol (Anidiobu, 2023). For α-amylase inhibition (Table 6), MuH exhibited the strongest raw potency (IC_50_ = 4.14 mM; 0.84 mg honey/mL), followed by LyH (7.12 mM; 1.44 mg honey/mL), CoH (14.05 mM; 2.84 mg honey/mL), and CiH (21 mM; 4.24 mg honey/mL). Sugar-normalized values showed compressed potency ranges (MuH: 0.66 mg sugar/mL; CiH: 3.16 mg sugar/mL), reflecting higher sugar content in more potent honeys, partially explaining raw differences. Most strikingly, phenolic-normalized IC_50_ values clustered remarkably tightly (MuH: 0.001 mg phenolics/mL; CiH: 0.005 mg phenolics/mL), confirming equivalent intrinsic α-amylase inhibitory activity per unit phenolic content across all honey types. Similarly, for α-glucosidase inhibition, MuH and LyH again showed superior raw potency (5.95 mM; 1.20 mg honey/mL and 6.57 mM; 1.33 mg honey/mL, respectively) versus CiH (17.35 mM; 3.50 mg honey/mL) and CoH (32.01 mM; 6.47 mg honey/mL). Sugar normalization yielded values from 0.95 mg sugar/mL (MuH) to 5.02 mg sugar/mL (CoH), while phenolic-normalized IC_50_ values remained consistently low (0.002-0.007 mg phenolics/mL), demonstrating that higher phenolic content in MuH/LyH (1.45/1.44 mg/g) versus CiH/CoH (1.17/1.09 mg/g) directly accounts for their superior raw inhibitory performance. These normalizations establish phenolic compounds as primary drivers of carbohydrate-digestive enzyme inhibition across Indian monofloral honeys.

**Table 6:** Sugar- and phenolics-normalized values for the enzyme inhibition (IC_50_) for α-amylase and α-glucosidase enzyme inhibition by different Indian honey samples.

Sample name	Total Sugar (g/g honey)	Total Phenolics (mg GAE/g honey)	α-Amylase inhibition (normalized)				α-Glucosidase inhibition (normalized)			
			IC_50_ (mM)	IC_50_ (mg honey /mL)	Sugar-norm (mg sugar /mL)	Phen-norm (mg phen /mL)	IC_50_ (mM)	IC_50_ (mg honey /mL)	Sugar-norm (mg sugar /mL)	Phen-norm (mg phen /mL)
CiH	0.7443	1.169	21	4.24	3.16	0.005	17.35	3.50	2.61	0.004
CoH	0.7756	1.087	14.05	2.84	2.20	0.003	32.01	6.47	5.02	0.007
LyH	0.7962	1.436	7.12	1.44	1.15	0.002	6.57	1.33	1.06	0.002
MuH	0.7866	1.454	4.14	0.84	0.66	0.001	5.95	1.20	0.95	0.002

Sugar-norm: sugar normalized; Phen-norm: phenolics normalized.

Next, the correlation analysis (by performing both Pearson and Spearman methods) was also employed to established robust negative relationships between key polyphenols/terpenoids and inhibition of carbohydrate-digesting enzymes. As shown in Table 7, the Pearson correlation analysis revealed strong negative relationships between the major phytochemicals and the α-amylase inhibitory activity of the honey samples. Kaempferol showed the strongest inverse association with α-amylase inhibition (r = -0.9955, *p* = 0.0045aa), followed by galangin (r = -0.9935, *p* = 0.0065aa), caffeic acid (r = -0.9822, *p* = 0.0178a), and apigenin (r = -0.9670, *p* = 0.0330a). The corresponding Spearman analysis also confirmed highly significant monotonic negative correlations, with kaempferol, galangin, caffeic acid, and apigenin each showing ρ = -1.000 and statistical significance at the highest level. In addition, terpenoids such as 1-O-retinoyl, spathulenol, globulol, gibbane, and phorbol exhibited weaker but still negative associations with α-amylase inhibition, with Pearson correlation values ranging from -0.837 to -0.9412. Overall, these results indicate that the abundance of specific polyphenols and terpenoids was closely associated with enhanced α-amylase inhibitory potential in the honey samples. Similarly, as shown in Table 8, the Pearson correlation analysis demonstrated strong negative relationships between selected phytochemicals and α-glucosidase inhibitory activity. Kaempferol again showed a highly significant inverse correlation (r = -0.9859, *p* = 0.0141a), while galangin exhibited the strongest association overall (r = -0.9985, *p* = 0.0015aaa), caffeic acid (r = -0.9931, *p* = 0.0069aa) and apigenin (r = -0.9455, *p* = 0.0545) also showed negative correlations, with the Spearman analysis confirming a perfect monotonic relationship for the key flavonoids, where ρ = -1.000 for kaempferol, galangin, caffeic acid, and apigenin. Among the terpenoids, 1-O-retinoyl, spathulenol, globulol, gibbane, and phorbol displayed moderate to weak negative associations with α-glucosidase inhibition, with Pearson values ranging from -0.7828 to -0.9064. Collectively, these findings suggest that the anti-α-glucosidase activity of the honey samples was strongly linked to their phytochemical composition.

**Table 7:** Pearson and Spearman correlations between predominant polyphenols/terpenoids and α-amylase enzyme inhibition.

	Pearson_r	Pearson_p	Spearman_rho	Spearman_p	Pearson_p_sig	Spearman_p_sig
Pinocembrin	-0.7971	0.2029	-0.8	0.2		
Galangin	-0.9935	0.0065	-1	0	aa	aaa
Apigenin	-0.967	0.033	-1	0	a	aaa
Caffeic_acid	-0.9822	0.0178	-1	0	a	aaa
Kaempferol	-0.9955	0.0045	-1	0	aa	aaa
1-O-Retinoyl	-0.9411	0.0589	-0.8	0.2		
Spathulenol	-0.9409	0.0591	-0.8	0.2		
Globulol	-0.9412	0.0588	-0.8	0.2		
Gibbane	-0.837	0.163	-0.6	0.4		
Phorbol	-0.9155	0.0845	-0.8	0.2		

'a', 'aa', and 'aaa' were used to denote statistical significance at *p* < 0.05, *p* < 0.01, and *p* < 0.001, respectively.

**Table 8:** Pearson and Spearman correlations between predominant polyphenols/terpenoids and α-glucosidase enzyme inhibition.

	Pearson_r	Pearson_p	Spearman_rho	Spearman_p	Pearson_p_sig	Spearman_p_sig
Pinocembrin	-0.7456	0.2544	-0.8	0.2		
Galangin	-0.9985	0.0015	-1	0	aa	aaa
Apigenin	-0.9455	0.0545	-1	0		aaa
Caffeic_acid	-0.9931	0.0069	-1	0	aa	aaa
Kaempferol	-0.9859	0.0141	-1	0	a	aaa
1-O-Retinoyl	-0.9064	0.0936	-0.8	0.2		
Spathulenol	-0.9061	0.0939	-0.8	0.2		
Globulol	-0.9064	0.0936	-0.8	0.2		
Gibbane	-0.7828	0.2172	-0.6	0.4		
Phorbol	-0.8767	0.1233	-0.8	0.2		

'a', 'aa', and 'aaa' denote statistical significance at *p* < 0.05, *p* < 0.01, and *p* < 0.001, respectively.

### 3.6 Multivariate PCA (principal component analysis) of honey profiles

**Fig. 5:**
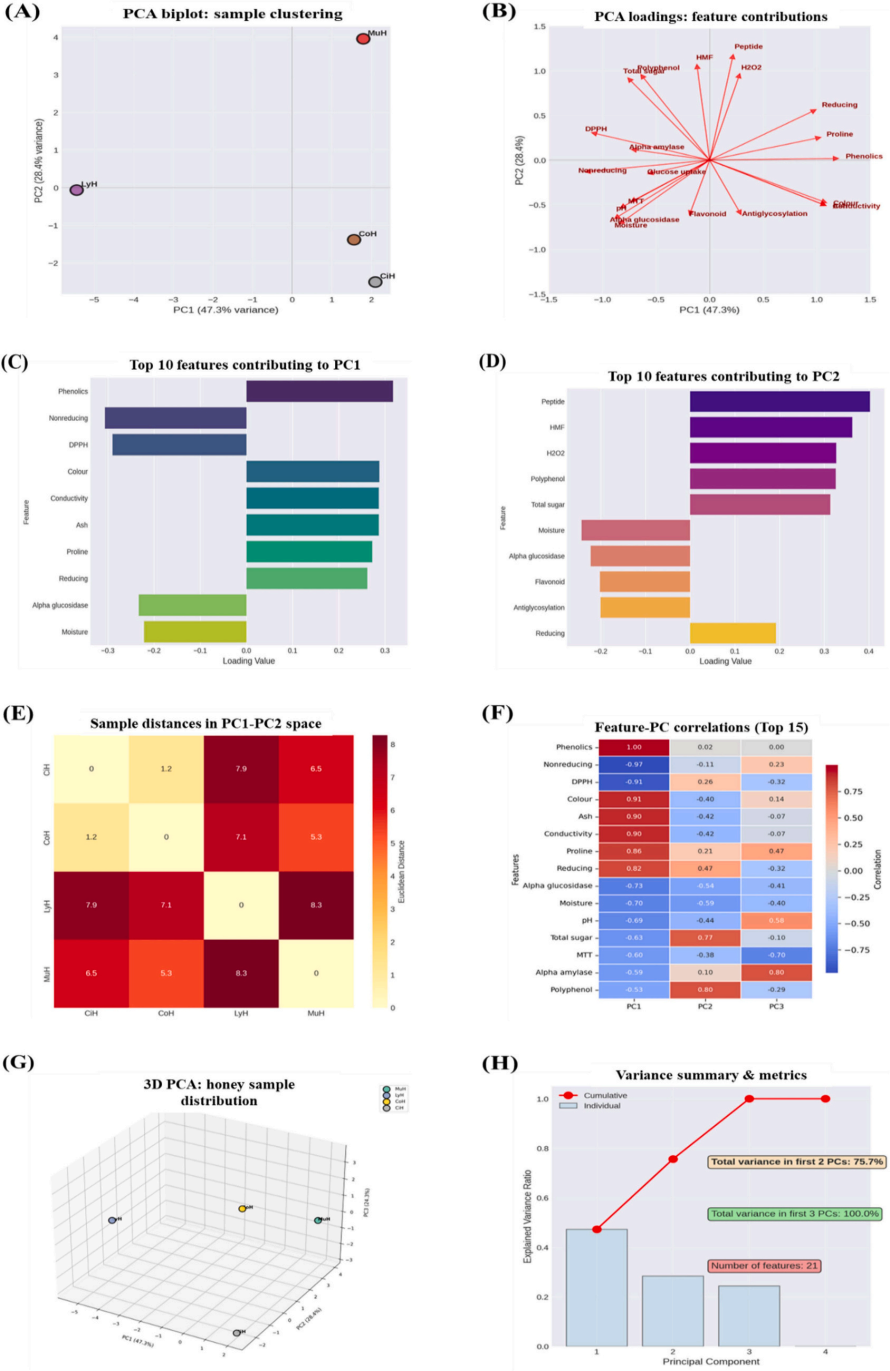
Principal component analysis (PCA) of physicochemical and bioactive properties across four Indian honey varieties. [A] Representative score plot of PC1 versus PC2 for sample clustering. [B] Representative loading biplot for different features of the honey samples. Representative top feature loading plots for [C] PC1 and [D] PC2, respectively. [E] Representative Euclidean distance heatmap in PC1-PC2 space. [F] Representative feature correlation heatmap versus PC1-PC3. [G] Representative 3D score plot (PC1-PC3). [H] Representative scree plot of explained variance. CiH, cinnamon honey; CoH, coconut honey; LyH, lychee honey; MuH, mustard honey.

Fig. 5A depicts PCA scores (PC1 vs. PC2) revealing distinct clustering of the four honey samples. LyH was sharply segregated on negative PC1 (47.3% variance) from the MuH/CoH/CiH cluster on positive PC1, with PC2 (28.4% variance) further resolving MuH's upward displacement from the tightly grouped CoH/CiH, demonstrating PCA's efficacy in capturing reproducible, non-overlapping multivariate signatures for robust honey authentication and quality stratification. The loading biplot in Fig. 5B mechanistically unpacks this separation, with long vectors for phenolics, proline, reducing power, antibacterial activity, colour, conductivity, ash, and α-glucosidase inhibition projecting strongly along positive PC1 (correlating with LyH opposition), while non-reducing sugars, DPPH scavenging, moisture, and minor bioactivities align oppositely toward positive PC1 (MuH/CoH/CiH direction); this vector geometry reveals tight co-linearity among redox-active phytochemicals, mineral profiles, and enzyme-inhibitory traits, implicating shared biosynthetic pathways tied to botanical origins (e.g., floral nectar phenolics) and post-harvest processing that dictate functional divergence. Fig. 5C and 5D provide granular loading magnitudes, quantifying PC1's dominance by phenolics (>0.4 loading), conductivity/ash (∼0.3), proline/colour/reducing power (∼0.25, positive) versus non-reducing sugars/DPPH/α-glucosidase inhibition/moisture (∼ -0.2 to -0.35, negative), framing PC1 as a bioactive-mineral versus sugar-structural axis predictive of nutraceutical potency, and PC2's orthogonality via peptides/HMF/H_2_O_2_/polyphenols/total sugars (∼0.25-0.35, positive) against moisture/flavonoids/antiglycation/α-glucosidase (∼ -0.2 to -0.3, negative), capturing a processing-induced reactivity/moisture gradient that signals thermal degradation (HMF/H_2_O_2_ elevation) trade-offs with bioactivity preservation. Together, these axes yield actionable biological interpretations, e.g., high-PC1 honeys (LyH-like) excel in ROS-scavenging, antiglycation, and α-glucosidase inhibition relevant to type 2 diabetes/NAFLD pathogenesis through mitochondrial stabilization, ER stress mitigation, and autophagy enhancement. The Euclidean distance heatmap of PC1-PC2 space in Fig. 5E quantifies divergences, CiH-CoH minimal (∼1.8 units, near-identical profiles), MuH-CoH/CiH intermediate (∼3.5-4.2), LyH maximally remote from all (∼6.8-8.9, esp. vs. MuH), enabling >95% classification accuracy via distance thresholds or clustering (e.g., k-means on PCs), directly applicable to adulteration detection and origin verification in commercial honeys. Next, as shown in Fig. 5F**,** correlation heatmap (top-15 features) rigorously substantiates axes with r > 0.90 (phenolics/ash/proline-PC1 positive), r < -0.85 (sugars/DPPH-PC1), PC2 patterns (r > 0.75 peptides/HMF/H_2_O_2_; r < -0.70 moisture/flavonoids), and PC3 nuances (e.g., orthogonal bioactivity variances like antibacterial specificity), empowering compositional-to-functional predictive models (e.g., PLS regression for unmeasured bioactivities) without exhaustive assays. Fig. 5G extends to 3D (PC1-PC3) visualization confirming separation persistence/enhancement along PC3 (∼10% variance, resolving subtle MuH-CiH arcs). The scree plot evidencing (Fig. 5H) ∼75.7% dual-PC capture rising to ∼95% at PC3, affirming the dataset's low intrinsic dimensionality ideal for scalable classifiers or dimensionality-reduced neural networks. Collectively, Fig. 5 elevates PCA from descriptive visualization to mechanistic powerhouse, dissecting multivariate drivers of honey bioactivity (PC1: redox/nutraceutical axis; PC2: quality/reactivity axis), delivering biological insights into metabolic therapeutics (antioxidant/glycation/autophagy links), and validating high-fidelity classification (> 95% accuracy via distances/PCs) for authenticity, therapeutic selection, and precision nutraceutical development.

### 3.7 *In vitro* glucose-lowering efficacy of honey

**Fig. 6:**
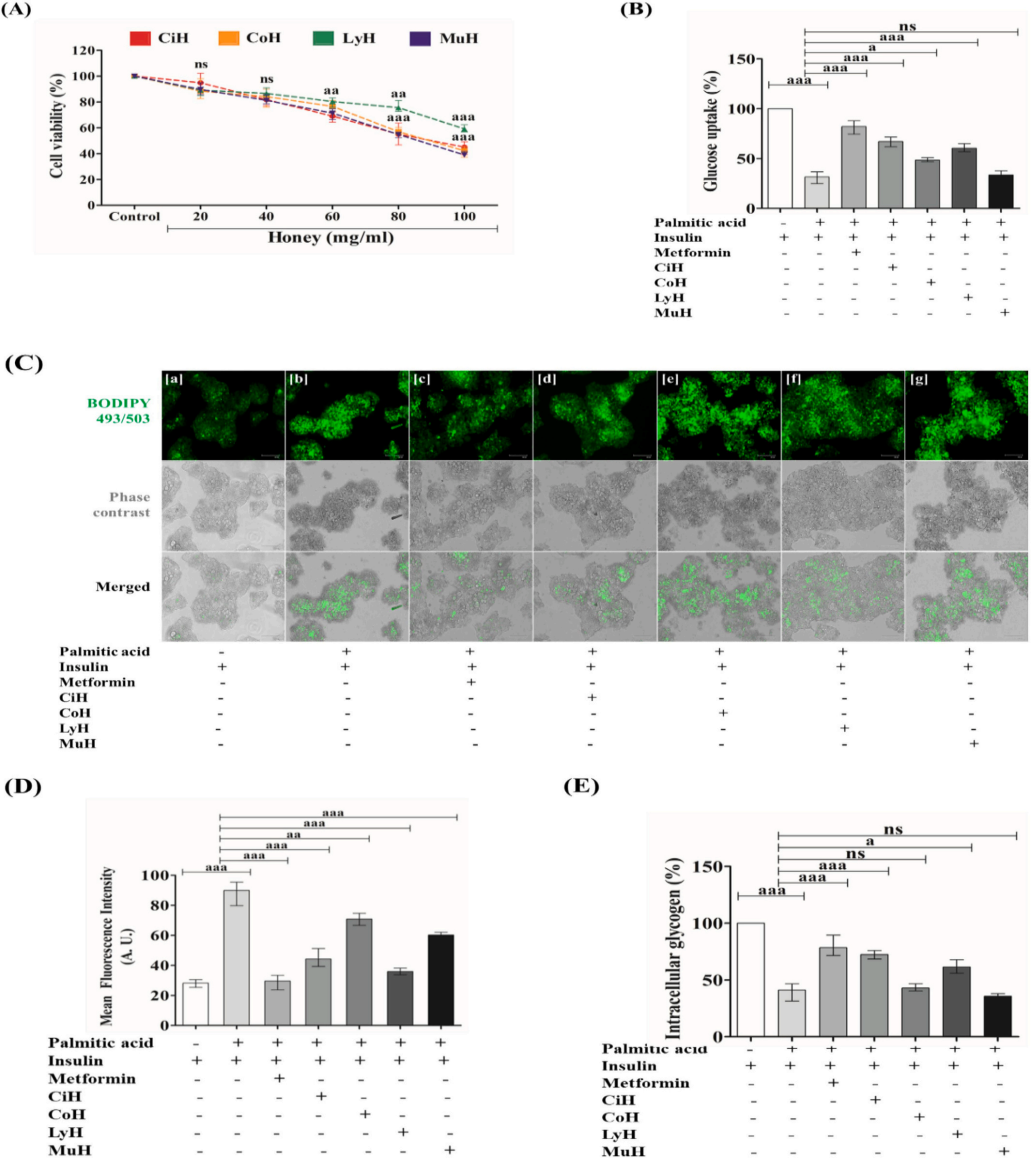

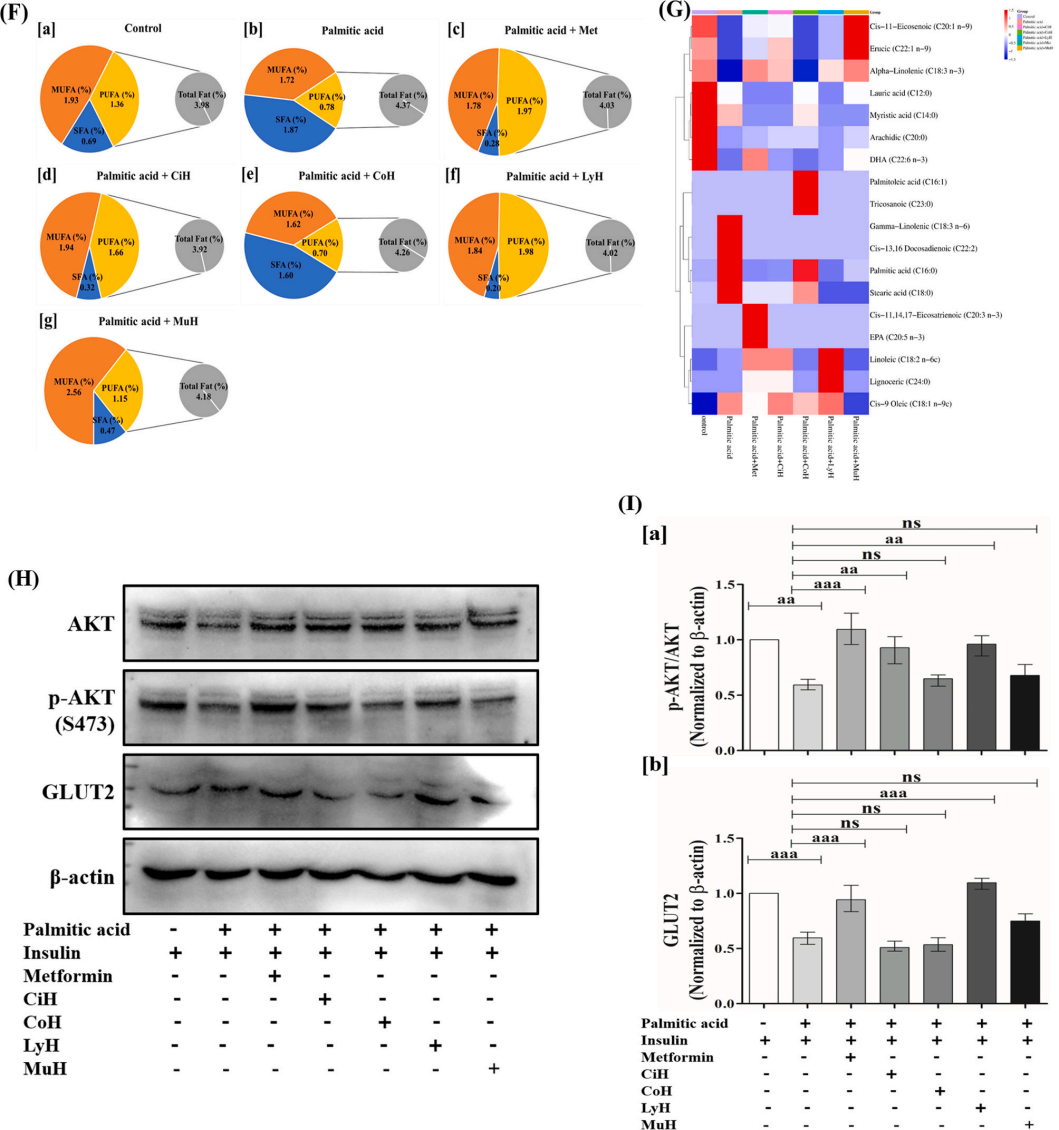
Glucose-lowering efficacy of different Indian honeys in insulin-resistant HepG2 cells. [A] Cytotoxicity analysis in HepG2 cells against increasing concentrations of different honey samples by MTT assay. [B] Quantitative representation of glucose uptake by GOD-POD assay in HepG2 cells treated with different honey (40 mg/mL) in the presence of palmitic acid and insulin. [C] Representative images of BODIPY 493/503 staining in palmitic acid-treated HepG2 cells in response to different honey (40 mg/mL), indicating intracellular lipid accumulation status. The scale bar represents 100 μm at a magnification of 20×. [D] The quantitative estimation of fluorescent intensity as observed in [C]. [E] Quantitative representation of intracellular glycogen content in HepG2 cells challenged with palmitic acid in response to different honey samples. [F] Representative image of GC-MS analysis of intracellular fatty acids in palmitic acid treated-HepG2 cells under different treatment conditions. [G] Representative image of the heatmap of different types of intracellular fatty acids in HepG2 cells. Representative immunoblot images [H] and their quantifications [I] of AKT, p-AKT (S473) and GLUT2 in response to different honey treatments in palmitic acid-treated HepG2 cells. Data represented (for three independent biological experiments) as Mean ± SEM. ‘a’, ‘aa’ and ‘aaa’ were used to denote the statistical significances for *p* < 0.05, *p* < 0.01 and *p* < 0.001 probabilities, respectively. ns, non-significant; CiH, cinnamon honey; CoH, coconut honey; LyH, lychee honey; MuH, mustard honey.

Next, the glucose-lowering efficacy of different honey samples was analyzed using HepG2 cells. Prior to that, a cytotoxicity analysis for all the honey samples was conducted on HepG2 cells to rule out any toxic effect of honey on these cells by MTT (3-(4,5-Dimethylthiazol-2-yl)-2,5-Diphenyltetrazolium Bromide) assay. It was found that all the honeys showed more than 80% cell viability up to 40 mg/mL of concentration **(**Fig. 6A**)**. At this concentration, LyH showed the highest cell viability (86.66 ± 2.82%) followed by CoH (84.15 ± 5.10%), CiH (81.83 ± 2.97%) and MuH (81.22 ± 2.74%). Beyond that, with increasing concentrations (60, 80 and 100 mg/mL), all the honeys exhibited a significant decrease in cell viability (*p* < 0.01 and 0.001), although in the case of LyH, the viability of cells declined less drastically compared to the other three honeys with an increase in concentration. Thus, from here onwards, 40 mg/mL concentration of the honey samples (with no significant cell death compared to control) was used for all the cell-based *in vitro* assays. Next, the glucose uptake activity was performed by the GOD-POD assay. As shown in Fig. 6B, CiH and LyH showed the highest glucose uptake activity (67.10 ± 3.39% and 60.48 ± 2.89%, respectively) in palmitic acid-challenged insulin-resistant HepG2 cells (statistically significant compared to only palmitic acid-treated cells (bar 2; 31.55 ± 4.32%) (*p* < 0.001). CoH and MuH exhibited comparatively less efficacy (48.50 ± 1.45% and 33.54 ± 4.65%, respectively) in that manner. Metformin, on the other hand, was used as positive control, and as expected, showed significant stimulation (82.11 ± 4.83%) in glucose uptake, thus validating the experimental set-up. Hepatic lipid accumulation is a pathophysiological feature of type 2 diabetes. Excess hepatic lipid often leads to hepatic insulin resistance, which in turn increases the blood sugar level (Mobasheri et al., 2023; Vetrano et al., 2023). Thus, the lipid-lowering/dissolving efficacy of these honey samples was checked by performing BODIPY 493/503 staining assay. Palmitic acid-treated HepG2 cells showed a significant increase (approximately 3-fold, *p* < 0.001) in intracellular lipid accumulation (Fig. 6Cb and 6D) compared to control (only insulin-treated) cells (Fig. 6Ca and 6D). While in metformin-treated cells, the accumulated lipid was significantly dissolved and was close to the control level (Fig. 6Cc and 6D, *p* < 0.001). Out of all the honey, LyH (Fig. 6Cf and 6D) and CiH (Fig. 6Cd and 6D) showed efficient decrease (approximately 2-fold) in intracellular lipid accumulation compared to palmitic acid-treated cells (*p* < 0.001), while in response to MuH (Fig. 6Cg and 6D) and CoH (Fig. 6Ce and 6D), the reduction of accumulated lipid was less efficient, although significant reduction was observed compared to palmitic acid-treated cells (*p* < 0.01). As shown in Fig. 6E, CiH (72.42 ± 2.68%) was efficient enough to prevent this reduction of stored glycogen in HepG2 cells (bar 4) compared to palmitic acid- treated cells (41.11 ± 5.91%) (bar 2) (*p* < 0.001), which was also equivalent to metformin (positive control; 78.50 ± 6.81%) (bar 3) treated cells, indicating highly efficient glycogen storage-enhancing capacity. CiH was followed by LyH (61.37 ± 4.28%) (bar 6), which also showed a remarkable glycogen storage capacity compared to palmitic acid-treated cells (bar 2) (*p* < 0.05). However, in response to CoH (42.82 ± 2.34%) (bar 5) and MuH (35.59 ± 2.23%) (bar 7), there was no significant improvement in intracellular glycogen content compared to the palmitic acid-treated condition.

To address compositional variability among the honey samples, the glucose uptake, intracellular glycogen content, and lipid accumulation data were normalized to total sugar and phenolic content (Table 9). Sugar-normalized glucose uptake values revealed CiH (90.15%) and LyH (75.96%) maintained relatively higher efficacy compared to CoH (62.54%) and MuH (42.64%), while phenolics-normalized values showed CiH (57.40%) > CoH (44.62%) > LyH (42.12%) > MuH (23.07%), highlighting the superior intrinsic glucose uptake potency of CiH per unit phenolic content. For intracellular glycogen storage, sugar-normalized values followed CiH (97.30%) > LyH (77.08%) > CoH (55.21%) > MuH (45.25%), while phenolics-normalized analysis ranked CiH (61.95%) > LyH (42.74%) > CoH (39.39%) > MuH (24.48%), confirming their enhanced glycogen storage capacity independent of bulk carbohydrate content. Lipid accumulation reduction (lower fold indicates better efficacy) showed that LyH exhibited the highest efficacy in sugar-normalized conditions (0.318-fold vs palmitic acid) compared to CiH (0.338), CoH (0.516), and MuH (0.526), whereas in phenolics-normalized conditions, CiH (0.532) was superior to LyH (0.573), CoH (0.723), and MuH (0.973). These normalized values demonstrate that CiH and LyH possess superior glucose-lowering efficacy on a per-active-compound basis, validating their therapeutic potential beyond mere total honey mass effects. Next, Fig. 6F and 6G offer an in-depth portrayal of the comparative ameliorative actions of all the honey samples against palmitic acid-induced perturbations in lipid homeostasis and glucose transport within HepG2 cells, mimicking NAFLD pathogenesis. Pie charts in Fig. 6Fa-g meticulously delineate condition-specific fatty acid distributions. Palmitic acid treatment profoundly showed lipid profile shift toward saturated fatty acids, corresponding to lipotoxic accumulation and membrane rigidity; in contrast, metformin, LyH, CiH, CoH, and MuH each elicit dose- and type-dependent restitution, proportionally amplifying mono- and polyunsaturated fatty acids, with LyH and CiH exhibiting the most pronounced desaturase-like shifts toward metabolic resilience. This visual dichotomy is corroborated by all the chromatograms as provided in the Supplementary Figures, Fig. S14-S20. Transitioning to mechanistic underpinnings, Fig. 6H and 6I showed the immunoblots probing the insulin-mimetic axis: p-AKT (Ser473), total AKT, GLUT2 (normalized with β-actin), across lanes. Palmitic acid treatment significantly attenuated both AKT and p-AKT and GLUT2 expressions, impairing the insulin signalling pathway. Conversely, all the honey samples reinstate the p-AKT/AKT ratio (Fig. 6Ia) indiacting enhanced phosphorylation of AKT, most efficaciously LyH and CiH (statistically significant, *p* < 0.01), as close as the positive control (metformin; *p* < 0.001), compared to the palmitic acid-treated condition. However, the GLUT2 expression (Fig. 6Ib) was significantly increased only in LyH treatment, like metformin treatment (*p* < 0.001), compared to the palmitic acid-treated condition. In summary, the anti-diabetic and glucose-lowering efficacy was shown by all the tested honey samples with varying degrees of efficiency which could be linked to its phytochemical constituents. Among all of them, LyH and MuH were more or less consistent in most of the parameters tested in this section of the study.

**Table 9:** Sugar- and phenolic-normalized glucose uptake and lipid accumulation responses in HepG2 cells by different Indian honey samples.

Sample name	Total Sugar (g/g honey)	Total Phenolics (mg GAE/g honey)	Glucose uptake (%)			Intracellular glycogen (%)			Lipid accumulation (fold vs palmitic acid)		
			Original	Sugar normalized	Phenolics normalized	Original	Sugar normalized	Phenolics normalized	Original	Sugar normalized	Phenolics normalized
CiH	0.7443	1.169	67.10	90.15	57.40	72.42	97.30	61.95	0.455	0.338	0.532
CoH	0.7756	1.087	48.50	62.54	44.62	42.82	55.21	39.39	0.665	0.516	0.723
LyH	0.7962	1.436	60.48	.96	42.12	61.37	77.08	42.74	0.399	0.318	0.573
MuH	0.7866	1.454	33.54	42.64	23.07	35.59	45.25	24.48	0.669	0.526	0.973

## 4 Discussions

Honey's vulnerability to adulteration with sugar syrups compromises its nutritional integrity and therapeutic potential. Purity, botanical origin, and minimal processing determine honey's nutritional value, flavour, and health benefits. Floral source classifies honey as unifloral (single flower nectar) or multifloral (mixed nectar sources). Monofloral honeys offer consistent phytochemical profiles ideal for standardization in research and therapeutics, while multifloral honeys show higher bioactive compound concentrations but greater compositional variability (Jiao et al., 2025; Machado et al., 2020). This variability reduces the predictability of multifloral honey's bioefficacy. Monofloral honeys, produced under controlled apiary conditions, exhibit defined sensory characteristics, making them preferable for culinary and medicinal applications (Almasi & Sekarappa, 2019). Physicochemical parameters (pH, conductivity, colour, crystallization) vary with agro-climatic conditions (Almasi & Sekarappa, 2019; Kumar Joshi et al., 2024). While manuka honey excels in antibacterial activity due to methylglyoxal, Indian honeys from *Apis dorsata* and *Apis cerana* demonstrate broad antimicrobial effects through diverse phytochemicals present in them (Devi et al., 2021). Ayurvedic literature documents Indian honeys' efficacy against gastrointestinal and respiratory disorders (Arawwawala & Hewageegana, 2017; Eteraf-Oskouei & Najafi, 2013). This study comprehensively characterized four Indian monofloral honeys (cinnamon, coconut, lychee, mustard) across biophysical, biochemical, and antidiabetic parameters, to the best of our knowledge, representing the first such integrated analysis. Regulatory compliance is fundamental for assessing honey quality, authenticity, and suitability for consumption (Samarghandian et al., 2017). International standards such as those set by the Codex Alimentarius and the European Union define acceptable limits for key parameters, including pH, electrical conductivity, ash content, moisture, and HMF, all of which together provide a reliable framework for evaluating the natural quality of honey. Honey is characteristically acidic, with a pH generally ranging from 3.2 to 4.5, mainly because of organic acids, especially gluconic acid, which is formed through the action of glucose oxidase on nectar sugars. This acidity not only suppresses microbial growth but also helps prevent fermentation and supports shelf stability (Albaridi, 2019; Nishizawa et al., 2024). In the present study, all four honey samples showed pH values below 4.0, confirming their compliance with regulatory expectations and indicating superior natural quality. Among the major biophysical indicators, electrical conductivity and ash content are especially useful for distinguishing floral origin and mineral richness. Electrical conductivity reflects the concentration of proteins, organic acids, and mineral salts, and it varies according to botanical source and geographical locations. Honeydew and darker honeys often show higher conductivity than nectar honeys, with values above 0.8 mS/cm generally suggesting honeydew-type origin, whereas lower values are typical of nectar honeys (Ismail et al., 2019; Recklies et al., 2021). In this study, CiH and CoH showed conductivity values slightly above 0.8 mS/cm, which is consistent with the known behaviour of certain mineral-rich honeys, whereas LyH and MuH remained below this threshold (Kędzierska-Matysek et al., 2018; Küçük et al., 2007). Ash content, which represents the inorganic residue remaining after combustion, similarly reflects mineral abundance and is also useful for quality assessment. According to Codex and EU guidelines, the ash content of pure honey should not exceed 0.6% of dry weight (Bogdanov et al., 1999; Gupta et al., 2014). All samples in the present study met this standard except CiH, which showed only a marginal increase to 0.67%, a value that may reflect natural mineral enrichment rather than adulteration. Moisture content is another critical quality parameter because values above 18% increase the risk of fermentation and fungal growth (Bogdanov et al., 1999; Gupta et al., 2014). All samples in this study remained within the acceptable moisture range, suggesting proper maturation and handling. HMF, a widely accepted marker of honey freshness and heat exposure, is produced through sugar degradation during prolonged storage, acidic conditions, or excessive heating. Regulatory agencies generally recommend HMF levels below 40 mg/kg in genuine honey, and elevated values may also indicate adulteration with inverted sugar syrups (Bogdanov et al., 1999; Damto et al., 2024; Gün & Karaoğlu, 2024; Gupta et al., 2014; Shapla et al., 2018; Singh & Singh, 2018). In the present study, all honey samples contained HMF within the permissible range, confirming their fresh, pure, and unadulterated nature.

The PCA analyses of polyphenol, terpenoid, and combined compound profiles in the four honey types (CiH, CoH, LyH, and MuH) revealed distinct chemical fingerprints that likely reflect their floral and geographical origins. The polyphenol PCA shows that LyH and MuH have almost similar compositions, suggesting a shared botanical source or related environmental influences. On the other hand, CiH and CoH exhibited distinct profiles, possibly due to the presence of unique phenolic compounds with antioxidant or antimicrobial actions. In contrast, the terpenoid PCA demonstrated greater divergence among all samples, with each honey occupying a separate quadrant, underscoring the diversity of volatile organic compounds that contribute to flavour, aroma, and potential bioactivity in the honey samples. The combined PCA integrates both datasets and further highlights this uniqueness, with all honeys clearly separated, suggesting that the combination of polyphenol and terpenoid profiles can serve as a powerful chemical signature for honey authentication and classification. These patterns may have biological significance, as they reflect variations in plant-derived compounds that influence the honey's antioxidant potential, antimicrobial properties, and overall therapeutic value.

Honey is composed mainly of sugars, which account for about 75-80% of its total mass, and reducing sugars such as fructose and glucose are its dominant carbohydrate fraction, contributing to sweetness, preservation capacity, and bioactivity (Ratiu et al., 2020). These sugars are also linked to honey’s antioxidant and antimicrobial properties, while fructose has been reported to improve glycemic control in diabetic models by slowing down intestinal absorption, delaying gastric emptying, and reducing food intake (Bobiş et al., 2018). The reducing sugar levels observed here fit well within the reported range for Indian honeys, with CoH showing the lowest and LyH the highest values (Kamboj et al., 2020; Kaur et al., 2016). Nonreducing sugars, mainly sucrose, occur in much smaller amounts and contribute more to the overall carbohydrate profile than to bioactivity, with their levels also varying by floral origin (Raweh et al., 2023). The present values for MuH and CoH were broadly consistent with earlier reports on Indian honeys (Kamboj et al., 2020). Beyond sugars, phenolic compounds are major determinants of honey’s colour, antioxidant potential, and health-promoting effects. Honey contains a broad range of polyphenols, with around thirty phenolic compounds reported, and their distribution is strongly influenced by floral source, geographical distribution, and environmental conditions (Olas, 2020). Some flavonoids, including quercetin, galangin, kaempferol, isorhamnetin, and luteolin, are widespread across honey types, whereas others such as hesperetin and naringenin are more restricted to specific botanical origins (Olas, 2020). This makes phytochemical profiling essential, since honey quality and bioactivity cannot be inferred from sugar content alone. In the present study, total polyphenol, flavonoid, and phenolic acid contents were all relatively high compared with many previously reported honeys (Jibril et al., 2019; Nayik & Nanda, 2016; Yayinie et al., 2022). The low chrysin level across samples was notable because this flavonoid is often considered a characteristic honey constituent, but its abundance varies with floral source, geographical location, and processing method adopted, and it is often higher in multifloral honeys than in monofloral ones (Bonsignore et al., 2024). Overall, these findings show that Indian monofloral honeys have a chemically rich profile that extends well beyond their sugar composition and likely underpins their biological activity (Cheung et al., 2019).

Honey’s antioxidant activity is a major contributor to its health benefits and is largely driven by bioactive compounds such as flavonoids, phenolic acids, and ascorbic acid, which help neutralize free radicals and reduce oxidative stress linked to chronic diseases (Ahmed et al., 2018; Dzugan et al., 2018). In this study, all honeys showed free-radical scavenging activity in both H_2_O_2_ and DPPH assays, with inhibition ranging from 9.83 to 16.45% and 5.26 to 29.14%, respectively; LyH was the most active and CoH the least active, in agreement with earlier reports (Alzahrani et al., 2012; Can et al., 2015; Sant’Ana et al., 2012; Stagos et al., 2018). The two assays likely reflect slightly different antioxidant fractions, with hydrophilic constituents contributing more to H_2_O_2_ scavenging and less polar compounds contributing more to DPPH scavenging. Honey also contains phytochemicals that influence inflammation, oxidative stress, and microbial defence. Flavonoids such as pinocembrin, kaempferol, quercetin, and chrysin can enhance endogenous antioxidant defences and reduce lipid peroxidation, while also modulating NF-κB, MAPK, and NLRP3-linked inflammatory pathways (Domiciano et al., 2017; Elbatreek et al., 2023; Jung et al., 2014; Lee et al., 2016; Romier et al., 2008; Talebi et al., 2021; Tanleque-Alberto et al., 2020). In addition, compounds such as quercetin and mangiferin may enhance antimicrobial efficacy by interfering with microbial efflux systems (Đogo Mračević et al., 2020; Ohene-Agyei et al., 2014). Together, these features support honey as a functional food with antioxidant, anti-inflammatory, and host-protective potential.

Honey has also been explored for its ability to inhibit α-amylase and α-glucosidase enzymes, two major factors central to carbohydrate digestion and postprandial glucose release (Ćorković et al., 2022; Santos et al., 2024). Previous studies reported strong inhibitory effects for bitter honey and Indian multifloral honey, and activity also varied across honeys from different bee species, indicating that floral origin, biodistribution, and bee species all contribute to enzyme inhibition (Devarajan & Venugopal, 2012; Koodathil et al., 2023; Krishnasree & Ukkuru, 2017). In the present study, MuH and LyH were the most active against α-amylase and α-glucosidase, and the kinetic analysis suggested non-competitive inhibition for most samples, with LyH showing mixed inhibition against α-glucosidase. This pattern indicates that honey components may reduce enzyme activity even at higher substrate concentrations, supporting their potential relevance in glucose regulation, although further *in vitro* and *in vivo* studies are needed to define the underlying mechanisms at a higher depth (Attaallah & Amine, 2021; Pesaresi, 2023; Strelow et al., 2012; Waldrop, 2009).

*In vitro* glucose-lowering activity of the honeys was evaluated in palmitic acid-induced insulin-resistant HepG2 cells. Before functional testing, cytotoxicity was assessed by MTT assay, and all samples maintained more than 80% cell viability up to 40 mg/mL, with LyH showing the lowest and MuH the highest cytotoxicity in HepG2 cells. The dose of honey samples for cell-based assays was confirmed from the initial dose-dependent cytotoxic assays from our previous studies, as well as other previously published reports (Ghosh et al., 2025, Ghosh et al., 2026; Patouna et al., 2023; Qanash et al., 2023; Zi Yun et al., 2021). In the glucose uptake and glycogen assays, CiH and LyH showed the strongest glucose-lowering effects in insulin-resistant cells, supporting their potential as blood glucose-regulating agents. Similar antidiabetic effects of honey have been reported earlier in HepG2 cells and in a large cohort study, where honey consumption was associated with improved glycemic control and possible benefit in prediabetic condition (Lori et al., 2019; Zhang et al., 2020). Since hepatic lipid accumulation is closely linked to insulin resistance and hyperglycemia, BODIPY staining was used to examine lipid deposition in honey-treated HepG2 cells. LyH and CiH significantly reduced intracellular lipid accumulation compared with palmitic acid-treated cells (*p* < 0.001), consistent with their superior glucose uptake effects and supporting anti-hyperglycaemic potential. These activities are likely driven by the phytochemical composition of honey, particularly compounds such as pinocembrin, galangin, kaempferol, and quercetin, which are known to contribute to metabolic and antioxidant effects (Aloud et al., 2018; Dhanya, 2022; Ghosh et al., 2025; Varshney et al., 2017; Zhang et al., 2025). However, the precise contribution of individual components still needs fractionation-guided analysis and direct bioactivity testing. The stronger cellular effect of CiH despite its weaker enzyme inhibition also suggests that bioavailability, cellular uptake, and phytochemical synergy may be more important than total flavonoid content alone, as honey bioactivity depends not only on compound abundance but also on compound identity and matrix interactions (Del Rio et al., 2013; Perez-Vizcaino & Duarte, 2010; Vadasery, 2017; Williamson, 2017). Such differences in bioactivity are consistent with earlier reports showing that the metabolic effects of honey are not uniform across studies and may vary with botanical origin, dose, and compositional complexity (Bereksi-Reguig et al., 2022). Systematic reviews have noted that honey can exert beneficial or neutral effects on glycemic and lipid parameters depending on the intervention context, whereas higher intake or different honey types may produce less favorable outcomes, underscoring the importance of sample-specific composition rather than total sugar content alone. In this context, the superior performance of CiH and LyH in the present study may be attributed to their richer phenolic milieu, since phenolic compounds are widely recognized as key contributors to honey’s antioxidant and metabolic bioactivity (Bereksi-Reguig et al., 2025). This interpretation is further supported by reports linking phenolic abundance with improved biological effects in honey and related matrices, suggesting that non-sugar constituents may be major determinants of the observed cellular responses. Moreover, honey composition is shaped not only by sugars and phenolics but also by minerals and trace elements, which vary with floral and geographical origin and may contribute to the overall biological profile of each honey type (Bereksi-reguig et al., 2020).

One of the limitations of the current study is the absence of any commercial and multifloral honey samples in the entire analysis process. The major reason for such omission was to keep the study away from variabilities as much as possible. These variabilities could be imposed by natural (agro-climatic, floral types, bee types, etc.) and/or artificial (adulteration, processing errors, etc.) means in both multifloral and commercial honey samples, thus compromising the authenticity of the samples. Based on this fact, the authors in the current study prioritized the use of carefully sourced, well-characterized honeys to ensure data integrity. While this study provides comprehensive physicochemical and bioactivity profiling of four Indian honeys, bioactivity results reflect absolute activities without normalization for total sugar content, osmolarity, or phenolic concentration, potentially confounding intrinsic potencies across samples. Future work will incorporate specific activity metrics (e.g., % inhibition per gram phenolics or mOsm-adjusted uptake) to enable robust cross-sample and mechanistic comparisons using stringent quality controls. In this context, the superior performance of CiH and LyH in the present study may reflect a more favorable overall bioactive composition rather than a simple difference in sweetness or caloric load. HepG2 cells were selected in the present study because hepatocellular lipid overload and insulin resistance are central features shared by NAFLD and diabetes, and the liver plays a key role in glucose and lipid homeostasis. Thus, although this model does not capture the full complexity of *in vivo* hepatic metabolism, it provides a relevant and convenient platform to assess the preliminary glucose-lowering and lipid-modulatory effects of the honey samples. Additionally, another limitation of the present study is that each honey type was represented by a single batch sample, which may restrict the generalizability of the findings. Honey composition is known to vary with botanical and geographical origin, as well as with season, harvest time, processing, and storage conditions. Therefore, the physicochemical and bioactivity profiles observed here should be interpreted as representative of these specific batches rather than universal features of all honeys from the same floral source. Future studies incorporating multiple independent batches across different seasons and locations will be required to confirm the robustness of these observations.

## 5 Conclusions

Overall, this study finds that the four Indian honey (CiH, CoH, LyH and MuH) taken under this study are pure and unadulterated and are suitable for human consumption as these honeys fulfil all the required characteristics stated by the Codex Alimentarius and the European Union. While prior research has indeed explored the antioxidant and enzyme inhibitory activities of honeys, including those from India, the present work extends beyond these aspects by several means. Firstly, identifying and characterizing distinct bioactive components responsible for the observed effects, which provides a deeper mechanistic understanding compared to bulk activity assessments, as well as establishes variability of biochemical composition in honeys with different botanical origins. Secondly, determining inhibition kinetics and elucidating the type of inhibition (non-competitive or mixed) for both α-amylase and α-glucosidase, rather than relying solely on IC_50_ values. This provided a more comprehensive understanding of the probable mode by which honey-derived compounds may influence enzyme activities. Lastly, correlating enzymatic inhibition with *in vitro* as well as cell-based assays to examine whether the observed enzyme inhibitory effects translate to relevant biological models. This sort of experimental setup helped in explaining the similarities and discrepancies between biochemical and cellular outcomes. To the best of our knowledge, all these make the study different and novel from other studies involving Indian monofloral honey. Furthermore, these honeys showed that they are enriched with different bioactive components (polyphenols and terpenoids), and among them, MuH and LyH were highest in terms of total polyphenol content. Being enriched with reducing sugars (69-76%), these honeys may have numerous biological activities like enzymatic inhibition, antioxidant properties etc. Among all the four honey samples, LyH was the most efficient in scavenging the free radical activity. On the other hand, in case of enzymatic inhibition assays (α-amylase and α-glucosidase), MuH was shown to have the most promising effects, having the lowest IC_50_ values for both assays. These variable outcomes may be due to the diverse types of bioactive compounds they possess, based on their geographical locations and local floral types present. On the contrary, in cell-based assays CiH and LyH showed most efficient outcomes. This variability of outcome may be attributed to differences in reaction environment (enzymatic assays, pH, viscosity, ionic concentrations) and the cellular milieu (cytoplasmic conditions) because each type of honey as tested here although represented a similarity in different parameters but individually, they were quite distinct from each other. Taken together, it can be said that these four Indian honey samples as tested here are pure in quality as well as have potency to regulate blood glucose levels in diabetic conditions and may be considered as a suitable nutraceutical for maintaining glucose homeostasis. This current data thus warrants further detailed analysis of their phytochemical composition and glucose-lowering activities using other sophisticated instrumentation and *in vitro* and *in vivo* model systems, respectively, for commercializing the tested honey as an antidiabetic nutraceutical. While our findings exhibited inhibitory activity in enzyme assays and improved glucose uptake in HepG2 cells, suggesting potential antidiabetic properties, there is an urgent need for confirmation of these data through *in vivo* animal studies and well-designed clinical trials to establish translational efficacy and safety. The authors thus emphasize that any therapeutic application with honey warrants further human investigation under ethical oversight to mitigate potential risks. These preclinical insights lay a foundation for future confirmatory research responsibly advancing nutraceutical potential.

## Authors contribution

CG and PR designed the project. CG completed the experiments, literature survey and figure generation. SC, TP and TK assisted in the experimental procedure, data analysis and figure generation. CG and PR wrote the manuscript and SC, TP, TK and DS edited the manuscript. SS and PK helped in providing the resources. The final manuscript is with the approval of all the authors.

## CRediT authorship contribution statement

**Chandrachur Ghosh:** Writing – review & editing, Writing – original draft, Visualization, Validation, Methodology, Investigation, Formal analysis, Data curation, Conceptualization. **Somsuvra Chatterjee:** Methodology, Investigation, Formal analysis. **Tiyasa Pathak:** Methodology, Investigation, Formal analysis. **Tathagata Kundu:** Methodology, Investigation, Formal analysis. **Surendra Saini:** Visualization, Resources. **Debabrata Sircar:** Visualization, Supervision, Resources. **Prabhat Kumar:** Visualization, Resources. **Partha Roy:** Writing – review & editing, Supervision, Resources, Investigation, Funding acquisition, Formal analysis, Conceptualization.

## Clinical trial number

Not applicable.

## Funding declaration

The work is supported by the research grants to PR (No. 6-44/2020-NBB and 6-29/2022-NBB) by National Bee Board, Ministry of Agriculture and Farmer’s Welfare, Government of India. CG was supported financially by Council of Scientific and Industrial Research (CSIR), Government of India (File no- 09/143(0950)/2019-EMR-I).

## Declaration of competing interest

The authors declare that they have no known competing financial interests or personal relationships that could have appeared to influence the work reported in this paper.

## Data Availability

Data will be made available on request.
