# Supplementary material for: Physicochemical, nutritional and biological characterization of different Indian monofloral honeys with special emphasis on their glucose-lowering efficacy
Source: Food Chem X. 2026 Jul 17;38:104198. doi: 10.1016/j.fochx.2026.104198 (PMC13470540; doi:10.1016/j.fochx.2026.104198)
Supplement: Supplementary file 1 [file mmc1.docx]

**Supplementary information to**

**Physicochemical, nutritional and biological characterization of different Indian monofloral honeys with special emphasis on their glucose-lowering efficacy**

Chandrachur Ghosh^1^, Somsuvra Chatterjee^1^, Tiyasa Pathak^1^, Tathagata Kundu^1^, Surendra Saini^1^, Debabrata Sircar^2^, Prabhat Kumar^3^, Partha Roy^1,4^*

*^1^Molecular Endocrinology Laboratory, Department of Biosciences and Bioengineering, Indian Institute of Technology Roorkee, Roorkee 247 667, Uttarakhand, India*

*^2^Plant Molecular Biology Laboratory, Department of Biosciences and Bioengineering, Indian Institute of Technology Roorkee, Roorkee 247 667, Uttarakhand, India*

*^3^National Bee Board, DA & FW, Ministry of Agriculture and Farmers Welfare, B Wing, 2nd Floor, Janpath Bhawan, Janpath, New Delhi – 110 001, India*

*^4^Center for Indian Knowledge Systems, Indian Institute of Technology Roorkee, Roorkee 247 667, Uttarakhand, India*

*Corresponding author: partha.roy@bt.iitr.ac.in ORCID ID: 0000-0003-1943-3079

**Chemicals used in this study**

# Palmitic acid (catalogue # TC386), MTT (catalogue # TC191), DAPI (catalogue # MB097), BSA (catalogue # TC194), DMSO (catalogue # RM5856) and α-amylase (catalogue # GRM638) were procured from Himedia (Mumbai, India). Heat inactivated fetal bovine serum (FBS) was purchased from Gibco (California, USA). 3,5-dinitrosalicylic acid (DNSA) (catalogue # 10253), acarbose (catalogue # 65457), α-glucosidase (catalogue # 75551) and p-nitrophenyl-β-D-galactopyranoside (pNPG) (catalogue # 96226) were purchased from SRL (Mumbai, India). All the other analytical reagents were purchased from Himedia (Mumbai, India) unless stated.

**Table S1:** **Melissopalynological analysis of pollen grains in honey**

| **Honey type** | **Dominant pollen (>45%)** | **Secondary pollen (16-45%)** | **Minor pollen (3-15%)** | **Trace (<3%)** | **Origin** |
| --- | --- | --- | --- | --- | --- |
| Cinnamon (CiH) | *Cinnamomum* spp.  (~77%) | *Schefflera* spp., *Terminalia* spp., *Aster* spp.  (~20%) | *Mangifera indica*,  *Citrus* spp., *Eucalyptus* spp., *Cocos nucifera*  (~7%) | *Lantana camara*, *Mimosa pudica*, *Fagopyrum esculentum*, *Psidium guajava*  (~2%) | Monofloral |
| Coconut (CoH) | *Cocos nucifera* (~81%) | *Coffea* spp., *Schefflera* spp., *Aster* spp.  (~16%) | *Syzygium* spp., *Terminalia* spp., *Brassica* spp., *Croton* spp.,  *Zea mays*,  *Mangifera indica*  (~11%) | Cajanus cajan, *Coriandrum sativum*  (~1%) | Monofloral |
| Lychee (LyH) | *Litchi chinensis* (~61%) | *Moringa oleifera*, *Mangifera indica*,  *Zea mays*  (~32%) | *Cajanus cajan*, *Psidium guajava*, Brassicaceae, Asteraceae, *Areca catechu*, *Cassia* spp., Fabaceae, Malvaceae, Solanaceae  (~4%) | *Coriandrum sativum*, *Syzygium cumini*, *Bombax ceiba*, *Callistemon* spp., *Allium* spp.  (~1%) | Monofloral |
| Mustard (MuH) | *Brassica* spp. (~78%) | *Moringa oleifera*, *Guizotia abyssinica,*  *Zea mays*, *Eucalyptus obliqua*, *Litchi chinensis*  (~17%) | *Cajanus cajan*, *Sesamum indicum*, *Psidium guajava*, *Mangifera indica*,  (~3%) | *Fagopyrum esculentum*, *Lantana camara*, *Mimosa pudica*, *Callistemon* spp. (~1%) | Monofloral |

**Table S2:** **Retention time of different polyphenols for various Indian honey samples as determined by LC-MS**

| **Polyphenols** | **CiH**  **Retention time (min)** | **CoH**  **Retention time (min)** | **LyH**  **Retention time (min)** | **MuH**  **Retention time (min)** |
| --- | --- | --- | --- | --- |
| **Fisetin** | 9.554 | 9.532 | 9.553 | 9.543 |
| **Naringenin** | 4.332 | 4.341 | 4.332 | 4.332 |
| **Galangin** | 8.824 | 8.831 | 8.817 | 8.815 |
| **Chrysin** | 3.132 | 3.129 | 3.133 | 3.132 |
| **Coniferaldehyde** | 7.561 | 7.542 | 7.563 | 7.562 |
| **Cyanidin 3-glucoside** | 7.585 | 7.583 | 7.582 | 7.580 |
| **Ellagic acid** | 8.002 | 8.001 | 8.004 | 8.004 |
| **Caffeic acid** | 4.320 | 4.323 | 4.330 | 4.332 |
| **Apigenin** | 7.671 | 7.675 | 7.670 | 7.675 |
| **Pinocembrin** | 6.221 | 6.221 | 6.223 | 6.214 |
| **Hesperidin** | 7.329 | 7.324 | 7.319 | 7.319 |
| **Luteolin** | 9.352 | 9.351 | 9.344 | 9.349 |
| **Scopoletin** | 8.682 | 8.683 | 8.691 | 8.691 |
| **Kaempferol** | 4.662 | 4.660 | 4.676 | 4.666 |
| **p-Coumaric acid** | 9.361 | 9.362 | 9.364 | 9.364 |
| **Quercetin** | 13.383 | 13.372 | 13.383 | 13.392 |
| **Genistein** | 5.365 | 5.363 | 5.365 | 5.347 |
| **Rutin** | 14.770 | 14.771 | 14.762 | 14.782 |
| **Sinapic acid** | 10.872 | 10.873 | 10.867 | 10.877 |
| **Syringaldehyde** | 7.693 | 7.695 | 7.685 | 7.685 |
| **Syringic acid** | 6.804 | 6.803 | 7.009 | 7.008 |
| **Taxifolin** | 9.579 | 9.578 | 9.598 | 9.597 |

**Table S3: Retention time of different terpenoids for various Indian honey samples as determined by LC-MS**

| **Terpenoids** | **CiH**  **Retention time (min)** | **CoH**  **Retention time (min)** | **LyH**  **Retention time (min)** | **MuH**  **Retention time (min)** |
| --- | --- | --- | --- | --- |
| **Bilobalide** | 7.654 | 7.623 | 7.625 | 7.622 |
| **Quassin** | 9.555 | 9.535 | 9.532 | 9.530 |
| **Alpha-amyrin** | 4.336 | 4.342 | 4.349 | 4.352 |
| **Globulol** | 8.812 | 8.820 | 8.825 | 8.828 |
| **Rimuene** | 3.110 | 3.113 | 3.115 | 3.117 |
| **Salvinorin** | 6.580 | 6.578 | 6.573 | 6.575 |
| **Cembrene** | 7.582 | 7.579 | 7.572 | 7.574 |
| **Scalerene** | 8.013 | 8.012 | 8.014 | 8.012 |
| **Betulin** | 4.356 | 4.358 | 4.351 | 4.353 |
| **Spathulenol** | 7.672 | 7.678 | 7.672 | 7.674 |
| **1-O-Retinoyl** | 6.223 | 6.225 | 6.220 | 6.223 |
| **Camphor** | 7.319 | 7.316 | 7.317 | 7.319 |
| **Ginkgolide** | 10.040 | 10.041 | 10.040 | 10.041 |
| **Laurenene** | 9.337 | 9.334 | 9.332 | 9.335 |
| **Cycloursan** | 8.697 | 8.693 | 8.691 | 8.692 |
| **Gibbane** | 4.678 | 4.675 | 4.677 | 4.678 |
| **Neral** | 9.364 | 9.362 | 9.363 | 9.361 |
| **Phorbol** | 13.387 | 13.381 | 13.382 | 13.384 |
| **Davana Ether** | 5.461 | 5.463 | 5.465 | 5.466 |
| **Chavicol** | 14.982 | 14.980 | 14.981 | 14.980 |
| **Sarisane** | 10.877 | 10.867 | 10.863 | 10.864 |
| **Lupeol** | 7.696 | 7.693 | 7.690 | 7.693 |
| **Isophorone** | 6.914 | 6.912 | 6.915 | 6.916 |
| **Apiol** | 9.552 | 9.550 | 9.552 | 9.554 |

**Table S4:** **MS/MS fragmentation patterns for different flavonoids for various Indian honey samples as determined by LC-MS**

| **Compound** | **Precursor [M+H]⁺ (m/z)** | **Fragmentor (V)** | **Quantifier (m/z)** | **Qualifier (m/z)** | **Collision Energy (eV)** |
| --- | --- | --- | --- | --- | --- |
| Fisetin | 287.1 | 120 | 269.1 | 241 | 15 |
| Naringenin | 273.1 | 115 | 153 | 119 | 15 |
| Galangin | 271.1 | 115 | 253 | 225 | 15 |
| Chrysin | 255.1 | 110 | 153 | 119 | 15 |
| Coniferaldehyde | 179.1 | 100 | 151 | 123 | 10 |
| Cyanidin‑3‑glucoside | 449.1 | 130 | 287.1 | 269.1 | 20 |
| Ellagic acid | 303 | 120 | 285 | 257 | 15 |
| Caffeic acid | 181 | 95 | 163 | 135 | 10 |
| Apigenin | 271.1 | 115 | 153 | 119 | 15 |
| Pinocembrin | 257.1 | 110 | 153 | 119 | 15 |
| Hesperidin | 611.2 | 150 | 449.1 | 303 | 20 |
| Luteolin | 287.1 | 120 | 269.1 | 241 | 15 |
| Scopoletin | 193.1 | 100 | 165 | 137 | 10 |
| Kaempferol | 287.1 | 120 | 269.1 | 241 | 15 |
| p‑Coumaric acid | 165 | 95 | 147 | 119 | 10 |
| Quercetin | 303 | 120 | 285 | 257 | 15 |
| Genistein | 271.1 | 115 | 153 | 119 | 15 |
| Rutin | 611.2 | 150 | 449.1 | 303 | 20 |
| Sinapic acid | 225.1 | 105 | 207 | 179 | 10 |
| Syringaldehyde | 183.1 | 100 | 155 | 127 | 10 |
| Syringic acid | 199.1 | 105 | 181 | 153 | 10 |
| Taxifolin | 305 | 120 | 287 | 259 | 15 |

**Table S5: MS/MS fragmentation patterns for different terpenoids for various Indian honey samples as determined by LC-MS**

| **Compound** | **Precursor [M+H]⁺ (m/z)** | **Fragmentor (V)** | **Quantifier (m/z)** | **Qualifier (m/z)** | **Collision Energy (eV)** |
| --- | --- | --- | --- | --- | --- |
| Bilobalide | 315.2 | 120 | 297.1 | 269.1 | 15 |
| Quassin | 357.2 | 130 | 339.1 | 311.1 | 15 |
| α‑Amyrin | 427.4 | 150 | 409.3 | 191.2 | 10 |
| Globulol | 223.2 | 110 | 205.1 | 177.1 | 15 |
| Rimuene | 273.2 | 120 | 255.2 | 227.2 | 15 |
| Salvinorin A | 433.2 | 130 | 415.2 | 387.2 | 20 |
| Cembrene | 273.2 | 115 | 255.2 | 227.2 | 15 |
| Scalerene | 273.2 | 115 | 255.2 | 227.2 | 10 |
| Betulin | 443.4 | 150 | 425.3 | 203.2 | 15 |
| Spathulenol | 223.2 | 110 | 205.1 | 177.1 | 15 |
| 1‑O‑Retinoyl‑β‑D‑glucopyranoside | 595.4 | 160 | 433.3 | 271.2 | 20 |
| Camphor | 153.1 | 100 | 135.1 | 107.1 | 15 |
| Ginkgolide B | 439.2 | 130 | 421.2 | 393.2 | 10 |
| Laurenene | 273.2 | 115 | 255.2 | 227.2 | 15 |
| Cycloursan | 273.2 | 115 | 255.2 | 227.2 | 10 |
| Gibbane | 273.2 | 115 | 255.2 | 227.2 | 15 |
| Neral | 153.1 | 100 | 135.1 | 107.1 | 15 |
| Phorbol | 347.2 | 130 | 329.2 | 301.2 | 20 |
| Davana ether | 207.2 | 110 | 189.1 | 161.1 | 10 |
| Chavicol | 137.1 | 95 | 119.1 | 91.1 | 10 |
| Sarisanine | 273.2 | 115 | 255.2 | 227.2 | 15 |
| Lupeol | 427.4 | 150 | 409.3 | 191.2 | 15 |
| Isophorone | 139.1 | 95 | 121.1 | 93.1 | 20 |
| Apiol | 207.1 | 110 | 189.1 | 161.1 | 10 |

**Table S6: Polyphenol profiling for Manuka honey as determined by LC-MS**

| **Polyphenol**  **compound** | **Retention time (RT)** | **Mean Conc.**  **(µg/kg)** | **SD** |
| --- | --- | --- | --- |
| 2-hydroxycinnamic acid | 11.12 | 124.75 | 15.54 |
| Acacetin | 11.097 | 107.49 | 14.55 |
| Apigenin | 11.878 | 63.99 | 11.13 |
| Betulin | 3.967 | 82.28 | 19.13 |
| Caffeic acid | 5.208 | 168.76 | 36.56 |
| Catechin | 9.098 | 210.76 | 19.51 |
| Chavicol | 4.613 | 349.89 | 29.24 |
| Chlorogenic acid | 4.886 | 58.03 | 1.62 |
| Chrysin | 10.148 | 46.94 | 7.01 |
| Epicatechin | 8.282 | 148.47 | 11.63 |
| Ferrulic acid | 5.722 | 176.34 | 20.2 |
| Galangin | 10.372 | 59.58 | 13.13 |
| Gallic acid | 4.61 | 96.64 | 15.96 |
| Genistein | 15.849 | 72.85 | 8.46 |
| Hesperetin | 14.091 | 108.42 | 1.92 |
| Kaempferol | 11.434 | 116.4 | 78.15 |
| Luteolin | 11.139 | 165.05 | 10.22 |
| Myricetin | 15.397 | 89.11 | 13.7 |
| Naringenin | 10.84 | 117.03 | 15.15 |
| Pinobanksin | 12.134 | 157.22 | 16.37 |
| Pinocembrin | 15.855 | 84.09 | 4.22 |
| Quercetin | 15.217 | 71.22 | 10.78 |
| Rimuene | 14.348 | 140.89 | 13.87 |
| Rutin | 16.452 | 134.05 | 9.44 |
| Sinapic acid | 7.614 | 399.46 | 21.12 |
| Syringic acids | 6.448 | 184.76 | 4.04 |
| Tricetin | 12.296 | 38.62 | 4.67 |
| Vanillic acid | 4.033 | 108.95 | 16.33 |
| p-Coumaric acid | 4.024 | 94.64 | 17.27 |

**Table S7: Sugar profiling for Manuka honey as determined by HPLC**

| **Sugar Compound** | **Retention Time (RT)** | **Mean conc. (%)** | **SD** |
| --- | --- | --- | --- |
| Fructose | 1.804 | 44.438 | 3.908 |
| Glucose | 2.138 | 35.305 | 0.594 |
| Maltose | 5.53 | 0.903 | 0.464 |
| Sucrose | 4.04 | 3.909 | 0.532 |

**Table S8: Pairwise comparisons for biophysical parameters in Table 1 with FDR-corrected p-values and Cohen's d effect sizes**

| **Parameter** | **Comparison** | **Mean_1** | **SEM_1** | **Mean_2** | **SEM_2** | **Mean_Difference** | **t_statistic** | **p_value** | **Cohens_d** | **CI_95_Lower** | **CI_95_Upper** | **Effect_Size** | **p_FDR_adjusted** | **Significant_FDR** |
| --- | --- | --- | --- | --- | --- | --- | --- | --- | --- | --- | --- | --- | --- | --- |
| **pH** | CiH vs CoH | 3.58 | 0.004 | 3.79 | 0.02 | 0.21 | 10.296 | <0.001 | 8.41 | 3.39 | 13.43 | Large | <0.001 | Yes |
| **pH** | CiH vs LyH | 3.58 | 0.004 | 3.83 | 0.01 | 0.25 | 23.212 | <0.001 | 18.95 | 8.11 | 29.79 | Large | <0.001 | Yes |
| **pH** | CiH vs MuH | 3.58 | 0.004 | 3.54 | 0.02 | -0.04 | -1.961 | 0.121393 | -1.6 | -3.44 | 0.24 | Large | 0.174806 | No |
| **pH** | CoH vs LyH | 3.79 | 0.02 | 3.83 | 0.01 | 0.04 | 1.789 | 0.148148 | 1.46 | -0.34 | 3.26 | Large | 0.205128 | No |
| **pH** | CoH vs MuH | 3.79 | 0.02 | 3.54 | 0.02 | -0.25 | -8.839 | <0.001 | -7.22 | -11.6 | -2.83 | Large | <0.001 | Yes |
| **pH** | LyH vs MuH | 3.83 | 0.01 | 3.54 | 0.02 | -0.29 | -12.969 | <0.001 | -10.59 | -16.79 | -4.39 | Large | <0.001 | Yes |
| **Electrical conductivity (mS/cm)** | CiH vs CoH | 0.91 | 0.003 | 0.81 | 0.003 | -0.1 | -23.57 | <0.001 | -19.25 | -30.25 | -8.24 | Large | <0.001 | Yes |
| **Electrical conductivity (mS/cm)** | CiH vs LyH | 0.91 | 0.003 | 0.41 | 0.004 | -0.5 | -100 | <0.001 | -81.65 | -127.88 | -35.42 | Large | <0.001 | Yes |
| **Electrical conductivity (mS/cm)** | CiH vs MuH | 0.91 | 0.003 | 0.67 | 0.003 | -0.24 | -56.569 | <0.001 | -46.19 | -72.37 | -20.01 | Large | <0.001 | Yes |
| **Electrical conductivity (mS/cm)** | CoH vs LyH | 0.81 | 0.003 | 0.41 | 0.004 | -0.4 | -80 | <0.001 | -65.32 | -102.31 | -28.33 | Large | <0.001 | Yes |
| **Electrical conductivity (mS/cm)** | CoH vs MuH | 0.81 | 0.003 | 0.67 | 0.003 | -0.14 | -32.998 | <0.001 | -26.94 | -42.27 | -11.61 | Large | <0.001 | Yes |
| **Electrical conductivity (mS/cm)** | LyH vs MuH | 0.41 | 0.004 | 0.67 | 0.003 | 0.26 | 52 | <0.001 | 42.46 | 18.38 | 66.53 | Large | <0.001 | Yes |
| **Ash content (%)** | CiH vs CoH | 0.67 | 0.003 | 0.58 | 0.003 | -0.09 | -21.213 | <0.001 | -17.32 | -27.25 | -7.39 | Large | <0.001 | Yes |
| **Ash content (%)** | CiH vs LyH | 0.67 | 0.003 | 0.25 | 0.003 | -0.42 | -98.995 | <0.001 | -80.83 | -126.59 | -35.07 | Large | <0.001 | Yes |
| **Ash content (%)** | CiH vs MuH | 0.67 | 0.003 | 0.46 | 0.002 | -0.21 | -58.244 | <0.001 | -47.56 | -74.51 | -20.6 | Large | <0.001 | Yes |
| **Ash content (%)** | CoH vs LyH | 0.58 | 0.003 | 0.25 | 0.003 | -0.33 | -77.782 | <0.001 | -63.51 | -99.48 | -27.54 | Large | <0.001 | Yes |
| **Ash content (%)** | CoH vs MuH | 0.58 | 0.003 | 0.46 | 0.002 | -0.12 | -33.282 | <0.001 | -27.17 | -42.63 | -11.72 | Large | <0.001 | Yes |
| **Ash content (%)** | LyH vs MuH | 0.25 | 0.003 | 0.46 | 0.002 | 0.21 | 58.244 | <0.001 | 47.56 | 20.6 | 74.51 | Large | <0.001 | Yes |
| **Moisture content (%)** | CiH vs CoH | 14.66 | 0.4 | 13.33 | 0.4 | -1.33 | -2.351 | 0.078418 | -1.92 | -3.85 | 0.01 | Large | 0.117627 | No |
| **Moisture content (%)** | CiH vs LyH | 14.66 | 0.4 | 16.33 | 0.4 | 1.67 | 2.952 | 0.041879 | 2.41 | 0.31 | 4.51 | Large | 0.068529 | No |
| **Moisture content (%)** | CiH vs MuH | 14.66 | 0.4 | 11.66 | 1.08 | -3 | -2.605 | 0.059742 | -2.13 | -4.13 | -0.12 | Large | 0.093509 | No |
| **Moisture content (%)** | CoH vs LyH | 13.33 | 0.4 | 16.33 | 0.4 | 3 | 5.303 | 0.006074 | 4.33 | 1.4 | 7.26 | Large | 0.010933 | Yes |
| **Moisture content (%)** | CoH vs MuH | 13.33 | 0.4 | 11.66 | 1.08 | -1.67 | -1.45 | 0.220653 | -1.18 | -2.92 | 0.55 | Large | 0.273914 | No |
| **Moisture content (%)** | LyH vs MuH | 16.33 | 0.4 | 11.66 | 1.08 | -4.67 | -4.055 | 0.015413 | -3.31 | -5.77 | -0.85 | Large | 0.026422 | Yes |
| **Colour (mm)** | CiH vs CoH | 138.28 | 82 | 137.51 | 0.54 | -0.77 | -0.009 | 0.992958 | -0.01 | -1.61 | 1.59 | Negligible | 0.992958 | No |
| **Colour (mm)** | CiH vs LyH | 138.28 | 82 | 66.87 | 2 | -71.41 | -0.871 | 0.433105 | -0.71 | -2.36 | 0.94 | Medium | 0.505471 | No |
| **Colour (mm)** | CiH vs MuH | 138.28 | 82 | 110.78 | 2.94 | -27.5 | -0.335 | 0.754351 | -0.27 | -1.88 | 1.33 | Small | 0.798725 | No |
| **Colour (mm)** | CoH vs LyH | 137.51 | 0.54 | 66.87 | 2 | -70.64 | -34.099 | <0.001 | -27.84 | -43.68 | -12.01 | Large | <0.001 | Yes |
| **Colour (mm)** | CoH vs MuH | 137.51 | 0.54 | 110.78 | 2.94 | -26.73 | -8.942 | <0.001 | -7.3 | -11.73 | -2.87 | Large | <0.001 | Yes |
| **Colour (mm)** | LyH vs MuH | 66.87 | 2 | 110.78 | 2.94 | 43.91 | 12.349 | <0.001 | 10.08 | 4.16 | 16.01 | Large | <0.001 | Yes |
| **HMF (mg/kg)** | CiH vs CoH | 13.03 | 1.2 | 11.91 | 0.78 | -1.12 | -0.783 | 0.477627 | -0.64 | -2.28 | 1 | Medium | 0.537330 | No |
| **HMF (mg/kg)** | CiH vs LyH | 13.03 | 1.2 | 13.86 | 1.03 | 0.83 | 0.525 | 0.627436 | 0.43 | -1.19 | 2.05 | Small | 0.684476 | No |
| **HMF (mg/kg)** | CiH vs MuH | 13.03 | 1.2 | 11.62 | 1.1 | -1.41 | -0.866 | 0.435267 | -0.71 | -2.36 | 0.94 | Medium | 0.505471 | No |
| **HMF (mg/kg)** | CoH vs LyH | 11.91 | 0.78 | 13.86 | 1.03 | 1.95 | 1.509 | 0.205734 | 1.23 | -0.51 | 2.98 | Large | 0.271746 | No |
| **HMF (mg/kg)** | CoH vs MuH | 11.91 | 0.78 | 11.62 | 1.1 | -0.29 | -0.215 | 0.840243 | -0.18 | -1.78 | 1.43 | Negligible | 0.864250 | No |
| **HMF (mg/kg)** | LyH vs MuH | 13.86 | 1.03 | 11.62 | 1.1 | -2.24 | -1.486 | 0.211358 | -1.21 | -2.96 | 0.53 | Large | 0.271746 | No |

**Table S9: Pairwise comparisons for the nutritional parameters in Table 4 with FDR-corrected p-values and Cohen's d effect sizes**

| **Parameter** | **Comparison** | **Mean_1** | **SEM_1** | **Mean_2** | **SEM_2** | **Mean_Difference** | **t_statistic** | **p_value** | **Cohens_d** | **CI_95_Lower** | **CI_95_Upper** | **Effect_Size** | **p_FDR_adjusted** | **Significant_FDR** |
| --- | --- | --- | --- | --- | --- | --- | --- | --- | --- | --- | --- | --- | --- | --- |
| **Total sugar (%)** | CiH vs CoH | 74.43 | 1.2 | 77.56 | 1.76 | 3.13 | 1.469 | 0.215667 | 1.2 | -0.54 | 2.94 | Large | 0.294091 | No |
| **Total sugar (%)** | CiH vs LyH | 74.43 | 1.2 | 79.62 | 1.86 | 5.19 | 2.345 | 0.078972 | 1.91 | -0.02 | 3.85 | Large | 0.121495 | No |
| **Total sugar (%)** | CiH vs MuH | 74.43 | 1.2 | 78.66 | 0.55 | 4.23 | 3.204 | 0.032761 | 2.62 | 0.44 | 4.8 | Large | 0.059565 | No |
| **Total sugar (%)** | CoH vs LyH | 77.56 | 1.76 | 79.62 | 1.86 | 2.06 | 0.804 | 0.466220 | 0.66 | -0.99 | 2.3 | Medium | 0.575293 | No |
| **Total sugar (%)** | CoH vs MuH | 77.56 | 1.76 | 78.66 | 0.55 | 1.1 | 0.597 | 0.582930 | 0.49 | -1.14 | 2.11 | Small | 0.699516 | No |
| **Total sugar (%)** | LyH vs MuH | 79.62 | 1.86 | 78.66 | 0.55 | -0.96 | -0.495 | 0.646594 | -0.4 | -2.02 | 1.21 | Small | 0.746070 | No |
| **Reducing sugar (%)** | CiH vs CoH | 71.89 | 0.98 | 69.2 | 1.13 | -2.69 | -1.798 | 0.146508 | -1.47 | -3.27 | 0.33 | Large | 0.204744 | No |
| **Reducing sugar (%)** | CiH vs LyH | 71.89 | 0.98 | 76.48 | 1.32 | 4.59 | 2.792 | 0.049215 | 2.28 | 0.22 | 4.34 | Large | 0.084369 | No |
| **Reducing sugar (%)** | CiH vs MuH | 71.89 | 0.98 | 76.13 | 0.43 | 4.24 | 3.962 | 0.016651 | 3.23 | 0.8 | 5.67 | Large | 0.033302 | Yes |
| **Reducing sugar (%)** | CoH vs LyH | 69.2 | 1.13 | 76.48 | 1.32 | 7.28 | 4.19 | 0.013810 | 3.42 | 0.91 | 5.93 | Large | 0.028572 | Yes |
| **Reducing sugar (%)** | CoH vs MuH | 69.2 | 1.13 | 76.13 | 0.43 | 6.93 | 5.732 | 0.004588 | 4.68 | 1.59 | 7.77 | Large | 0.013717 | Yes |
| **Reducing sugar (%)** | LyH vs MuH | 76.48 | 1.32 | 76.13 | 0.43 | -0.35 | -0.252 | 0.813379 | -0.21 | -1.81 | 1.4 | Small | 0.871477 | No |
| **Non-reducing sugar (%)** | CiH vs CoH | 2.36 | 0.36 | 5.42 | 0.25 | 3.06 | 6.982 | 0.002214 | 5.7 | 2.1 | 9.3 | Large | 0.007814 | Yes |
| **Non-reducing sugar (%)** | CiH vs LyH | 2.36 | 0.36 | 3.19 | 0.9 | 0.83 | 0.856 | 0.440117 | 0.7 | -0.95 | 2.35 | Medium | 0.574066 | No |
| **Non-reducing sugar (%)** | CiH vs MuH | 2.36 | 0.36 | 2.2 | 1.6 | -0.16 | -0.098 | 0.926974 | -0.08 | -1.68 | 1.52 | Negligible | 0.940080 | No |
| **Non-reducing sugar (%)** | CoH vs LyH | 5.42 | 0.25 | 3.19 | 0.9 | -2.23 | -2.387 | 0.075382 | -1.95 | -3.89 | -0.01 | Large | 0.121495 | No |
| **Non-reducing sugar (%)** | CoH vs MuH | 5.42 | 0.25 | 2.2 | 1.6 | -3.22 | -1.988 | 0.117669 | -1.62 | -3.47 | 0.22 | Large | 0.173526 | No |
| **Non-reducing sugar (%)** | LyH vs MuH | 3.19 | 0.9 | 2.2 | 1.6 | -0.99 | -0.539 | 0.618305 | -0.44 | -2.06 | 1.18 | Small | 0.727418 | No |
| **Total polyphenols (µg/g)** | CiH vs CoH | 1168.53 | 32.56 | 1086.94 | 31.64 | -81.59 | -1.797 | 0.146733 | -1.47 | -3.27 | 0.34 | Large | 0.204744 | No |
| **Total polyphenols (µg/g)** | CiH vs LyH | 1168.53 | 32.56 | 1435.66 | 42.58 | 267.13 | 4.984 | 0.007578 | 4.07 | 1.27 | 6.87 | Large | 0.018187 | Yes |
| **Total polyphenols (µg/g)** | CiH vs MuH | 1168.53 | 32.56 | 1453.98 | 23.34 | 285.45 | 7.125 | 0.002051 | 5.82 | 2.16 | 9.48 | Large | 0.007691 | Yes |
| **Total polyphenols (µg/g)** | CoH vs LyH | 1086.94 | 31.64 | 1435.66 | 42.58 | 348.72 | 6.574 | 0.002772 | 5.37 | 1.93 | 8.8 | Large | 0.008754 | Yes |
| **Total polyphenols (µg/g)** | CoH vs MuH | 1086.94 | 31.64 | 1453.98 | 23.34 | 367.04 | 9.335 | <0.001 | 7.62 | 3.02 | 12.22 | Large | <0.001 | Yes |
| **Total polyphenols (µg/g)** | LyH vs MuH | 1435.66 | 42.58 | 1453.98 | 23.34 | 18.32 | 0.377 | 0.725125 | 0.31 | -1.3 | 1.92 | Small | 0.820896 | No |
| **Total flavonoids (µg/g)** | CiH vs CoH | 296.41 | 4.28 | 241.97 | 2.33 | -54.44 | -11.171 | <0.001 | -9.12 | -14.52 | -3.72 | Large | <0.001 | Yes |
| **Total flavonoids (µg/g)** | CiH vs LyH | 296.41 | 4.28 | 269.35 | 2.39 | -27.06 | -5.52 | 0.005258 | -4.51 | -7.52 | -1.5 | Large | 0.013717 | Yes |
| **Total flavonoids (µg/g)** | CiH vs MuH | 296.41 | 4.28 | 211.92 | 4.79 | -84.49 | -13.153 | <0.001 | -10.74 | -17.02 | -4.46 | Large | <0.001 | Yes |
| **Total flavonoids (µg/g)** | CoH vs LyH | 241.97 | 2.33 | 269.35 | 2.39 | 27.38 | 8.203 | 0.001203 | 6.7 | 2.58 | 10.81 | Large | 0.004812 | Yes |
| **Total flavonoids (µg/g)** | CoH vs MuH | 241.97 | 2.33 | 211.92 | 4.79 | -30.05 | -5.641 | 0.004860 | -4.61 | -7.66 | -1.55 | Large | 0.013717 | Yes |
| **Total flavonoids (µg/g)** | LyH vs MuH | 269.35 | 2.39 | 211.92 | 4.79 | -57.43 | -10.728 | <0.001 | -8.76 | -13.97 | -3.55 | Large | <0.001 | Yes |
| **Total phenolic acids (µg/g)** | CiH vs CoH | 682.37 | 2.53 | 651.76 | 3.8 | -30.61 | -6.705 | 0.002575 | -5.47 | -8.96 | -1.99 | Large | 0.008583 | Yes |
| **Total phenolic acids (µg/g)** | CiH vs LyH | 682.37 | 2.53 | 310.89 | 8.77 | -371.48 | -40.698 | <0.001 | -33.23 | -52.1 | -14.36 | Large | <0.001 | Yes |
| **Total phenolic acids (µg/g)** | CiH vs MuH | 682.37 | 2.53 | 670.63 | 3.08 | -11.74 | -2.945 | 0.042162 | -2.4 | -4.51 | -0.3 | Large | 0.074404 | No |
| **Total phenolic acids (µg/g)** | CoH vs LyH | 651.76 | 3.8 | 310.89 | 8.77 | -340.87 | -35.664 | <0.001 | -29.12 | -45.67 | -12.57 | Large | <0.001 | Yes |
| **Total phenolic acids (µg/g)** | CoH vs MuH | 651.76 | 3.8 | 670.63 | 3.08 | 18.87 | 3.858 | 0.018182 | 3.15 | 0.75 | 5.55 | Large | 0.034091 | Yes |
| **Total phenolic acids (µg/g)** | LyH vs MuH | 310.89 | 8.77 | 670.63 | 3.08 | 359.74 | 38.702 | <0.001 | 31.6 | 13.65 | 49.55 | Large | <0.001 | Yes |
| **Total protein content (mg/g)** | CiH vs CoH | 0.61 | 0.05 | 0.86 | 0.19 | 0.25 | 1.272 | 0.272144 | 1.04 | -0.67 | 2.74 | Large | 0.362859 | No |
| **Total protein content (mg/g)** | CiH vs LyH | 0.61 | 0.05 | 0.66 | 0.15 | 0.05 | 0.316 | 0.767644 | 0.26 | -1.35 | 1.87 | Small | 0.841960 | No |
| **Total protein content (mg/g)** | CiH vs MuH | 0.61 | 0.05 | 0.64 | 0.2 | 0.03 | 0.146 | 0.891338 | 0.12 | -1.48 | 1.72 | Negligible | 0.935762 | No |
| **Total protein content (mg/g)** | CoH vs LyH | 0.86 | 0.19 | 0.66 | 0.15 | -0.2 | -0.826 | 0.455125 | -0.67 | -2.32 | 0.97 | Medium | 0.575293 | No |
| **Total protein content (mg/g)** | CoH vs MuH | 0.86 | 0.19 | 0.64 | 0.2 | -0.22 | -0.797 | 0.469823 | -0.65 | -2.29 | 0.99 | Medium | 0.575293 | No |
| **Total protein content (mg/g)** | LyH vs MuH | 0.66 | 0.15 | 0.64 | 0.2 | -0.02 | -0.08 | 0.940080 | -0.07 | -1.67 | 1.54 | Negligible | 0.940080 | No |
| **Proline content (mg/g)** | CiH vs CoH | 1.98 | 0.21 | 3.38 | 0.18 | 1.4 | 5.062 | 0.007172 | 4.13 | 1.3 | 6.97 | Large | 0.017930 | Yes |
| **Proline content (mg/g)** | CiH vs LyH | 1.98 | 0.21 | 1.89 | 0.2 | -0.09 | -0.31 | 0.771797 | -0.25 | -1.86 | 1.35 | Small | 0.841960 | No |
| **Proline content (mg/g)** | CiH vs MuH | 1.98 | 0.21 | 2.94 | 0.13 | 0.96 | 3.887 | 0.017737 | 3.17 | 0.77 | 5.58 | Large | 0.034091 | Yes |
| **Proline content (mg/g)** | CoH vs LyH | 3.38 | 0.18 | 1.89 | 0.2 | -1.49 | -5.538 | 0.005199 | -4.52 | -7.54 | -1.5 | Large | 0.013717 | Yes |
| **Proline content (mg/g)** | CoH vs MuH | 3.38 | 0.18 | 2.94 | 0.13 | -0.44 | -1.982 | 0.118576 | -1.62 | -3.46 | 0.23 | Large | 0.173526 | No |
| **Proline content (mg/g)** | LyH vs MuH | 1.89 | 0.2 | 2.94 | 0.13 | 1.05 | 4.402 | 0.011675 | 3.59 | 1.01 | 6.18 | Large | 0.025018 | Yes |
| **H2O2 scavenging activity (%)** | CiH vs CoH | 12.94 | 1.23 | 9.83 | 0.49 | -3.11 | -2.349 | 0.078607 | -1.92 | -3.85 | 0.02 | Large | 0.121495 | No |
| **H2O2 scavenging activity (%)** | CiH vs LyH | 12.94 | 1.23 | 16.45 | 0.45 | 3.51 | 2.68 | 0.055232 | 2.19 | 0.16 | 4.21 | Large | 0.092053 | No |
| **H2O2 scavenging activity (%)** | CiH vs MuH | 12.94 | 1.23 | 13.11 | 0.51 | 0.17 | 0.128 | 0.904570 | 0.1 | -1.5 | 1.71 | Negligible | 0.935762 | No |
| **H2O2 scavenging activity (%)** | CoH vs LyH | 9.83 | 0.49 | 16.45 | 0.45 | 6.62 | 9.951 | <0.001 | 8.12 | 3.26 | 12.99 | Large | <0.001 | Yes |
| **H2O2 scavenging activity (%)** | CoH vs MuH | 9.83 | 0.49 | 13.11 | 0.51 | 3.28 | 4.638 | 0.009751 | 3.79 | 1.11 | 6.46 | Large | 0.021669 | Yes |
| **H2O2 scavenging activity (%)** | LyH vs MuH | 16.45 | 0.45 | 13.11 | 0.51 | -3.34 | -4.911 | 0.007982 | -4.01 | -6.79 | -1.23 | Large | 0.018420 | Yes |
| **DPPH scavenging activity (%)** | CiH vs CoH | 15.62 | 0.45 | 5.26 | 0.26 | -10.36 | -19.934 | <0.001 | -16.28 | -25.62 | -6.93 | Large | <0.001 | Yes |
| **DPPH scavenging activity (%)** | CiH vs LyH | 15.62 | 0.45 | 29.14 | 0.2 | 13.52 | 27.455 | <0.001 | 22.42 | 9.63 | 35.2 | Large | <0.001 | Yes |
| **DPPH scavenging activity (%)** | CiH vs MuH | 15.62 | 0.45 | 24.18 | 0.56 | 8.56 | 11.915 | <0.001 | 9.73 | 4 | 15.46 | Large | <0.001 | Yes |
| **DPPH scavenging activity (%)** | CoH vs LyH | 5.26 | 0.26 | 29.14 | 0.2 | 23.88 | 72.799 | <0.001 | 59.44 | 25.77 | 93.11 | Large | <0.001 | Yes |
| **DPPH scavenging activity (%)** | CoH vs MuH | 5.26 | 0.26 | 24.18 | 0.56 | 18.92 | 30.644 | <0.001 | 25.02 | 10.77 | 39.27 | Large | <0.001 | Yes |
| **DPPH scavenging activity (%)** | LyH vs MuH | 29.14 | 0.2 | 24.18 | 0.56 | -4.96 | -8.341 | 0.001129 | -6.81 | -10.98 | -2.64 | Large | 0.004812 | Yes |

**Enzyme kinetics raw data:**

The alpha-amylase inhibition raw data is given here.

The alpha-glucosidase inhibition raw data is given here.

**Figure S1: Quantitative analysis report of polyphenolic compounds in the CiH sample as determined by LC-MS-based chromatographic profiling.** The figure shows a representative sample chromatogram with retention time on the x-axis and signal intensity (counts) on the y-axis, followed by a summary table of the identified phenolic and flavonoid compounds, including their retention times, detector responses, and calculated concentrations (mcg/l). This report illustrates the separation and quantitation performance of the analytical method for multiple analytes within a single run, supporting the reliability of the polyphenol profiling employed in the study.

**Figure S2: Quantitative analysis report of polyphenolic compounds in the CoH sample as determined by LC-MS-based chromatographic profiling.** The figure shows a representative sample chromatogram with retention time on the x-axis and signal intensity (counts) on the y-axis, followed by a summary table of the identified phenolic and flavonoid compounds, including their retention times, detector responses, and calculated concentrations (mcg/l). This report illustrates the separation and quantitation performance of the analytical method for multiple analytes within a single run, supporting the reliability of the polyphenol profiling employed in the study.

**Figure S3: Quantitative analysis report of polyphenolic compounds in the LyH sample as determined by LC-MS-based chromatographic profiling.** The figure shows a representative sample chromatogram with retention time on the x-axis and signal intensity (counts) on the y-axis, followed by a summary table of the identified phenolic and flavonoid compounds, including their retention times, detector responses, and calculated concentrations (mcg/l). This report illustrates the separation and quantitation performance of the analytical method for multiple analytes within a single run, supporting the reliability of the polyphenol profiling employed in the study.

**Figure S4: Quantitative analysis report of polyphenolic compounds in the MuH sample as determined by LC-MS-based chromatographic profiling.** The figure shows a representative sample chromatogram with retention time on the x-axis and signal intensity (counts) on the y-axis, followed by a summary table of the identified phenolic and flavonoid compounds, including their retention times, detector responses, and calculated concentrations (mcg/l). This report illustrates the separation and quantitation performance of the analytical method for multiple analytes within a single run, supporting the reliability of the polyphenol profiling employed in the study.

**Figure S5: Quantitative analysis report of terpenoid compounds in the CiH sample as determined by LC-MS-based chromatographic profiling.** The figure shows a representative sample chromatogram with retention time on the x-axis and signal intensity (counts) on the y-axis, followed by a summary table of the identified phenolic and flavonoid compounds, including their retention times, detector responses, and calculated concentrations (mcg/l). This report illustrates the separation and quantitation performance of the analytical method for multiple analytes within a single run, supporting the reliability of the polyphenol profiling employed in the study.

**Figure S6: Quantitative analysis report of terpenoid compounds in the CoH sample as determined by LC-MS-based chromatographic profiling.** The figure shows a representative sample chromatogram with retention time on the x-axis and signal intensity (counts) on the y-axis, followed by a summary table of the identified phenolic and flavonoid compounds, including their retention times, detector responses, and calculated concentrations (mcg/l). This report illustrates the separation and quantitation performance of the analytical method for multiple analytes within a single run, supporting the reliability of the polyphenol profiling employed in the study.

**Figure S7: Quantitative analysis report of terpenoid compounds in the LyH sample as determined by LC-MS-based chromatographic profiling.** The figure shows a representative sample chromatogram with retention time on the x-axis and signal intensity (counts) on the y-axis, followed by a summary table of the identified phenolic and flavonoid compounds, including their retention times, detector responses, and calculated concentrations (mcg/l). This report illustrates the separation and quantitation performance of the analytical method for multiple analytes within a single run, supporting the reliability of the polyphenol profiling employed in the study.

**Figure S8: Quantitative analysis report of terpenoid compounds in the MuH sample as determined by LC-MS-based chromatographic profiling.** The figure shows a representative sample chromatogram with retention time on the x-axis and signal intensity (counts) on the y-axis, followed by a summary table of the identified phenolic and flavonoid compounds, including their retention times, detector responses, and calculated concentrations (mcg/l). This report illustrates the separation and quantitation performance of the analytical method for multiple analytes within a single run, supporting the reliability of the polyphenol profiling employed in the study.

**Figure S9: Quantitative analysis report of polyphenolic compounds in the Manuka honey sample as determined by LC-MS-based chromatographic profiling.** The figure shows a representative sample chromatogram with retention time on the x-axis and signal intensity (counts) on the y-axis, followed by a summary table of the identified phenolic and flavonoid compounds, including their retention times, detector responses, and calculated concentrations (mcg/l). This report illustrates the separation and quantitation performance of the analytical method for multiple analytes within a single run, supporting the reliability of the polyphenol profiling employed in the study.

**Figure S10: Quantitative analysis report of sugar compounds in the Manuka honey sample as determined by HPLC-based chromatographic profiling.** The figure shows a representative sample chromatogram with retention time on the x-axis and signal intensity (counts) on the y-axis, followed by a summary table of the identified sugars, including their retention times, area, and calculated concentrations (%). This report illustrates the separation and quantitation performance of the analytical method for multiple analytes within a single run, supporting the reliability of the sugar profiling employed in the study.

**Figure S11: Standard curve of Trolox for its H_2_O_2_ and DPPH scavenging activities.** The figure displays dose-response curves plotting the percentage inhibition of H_2_O_2_ **[A**] and DPPH **[B]** scavenging against increasing concentration of Trolox. Both curves demonstrate a concentration-dependent increase in antioxidant activities, with higher Trolox levels yielding greater scavenging effects, as evidenced by the increasing % inhibition values. The linear trends suggest Trolox's efficacy in neutralizing both H_2_O_2_ and DPPH radicals, providing a reference for quantifying antioxidant capacity in comparative assays. Data are mean ± SEM of three independent experiments.

**Figure S12: Shapiro-Wilk test (for normality assessment) for Figure 6C.** The Shapiro-Wilk test results in the Figure indicate that all groups [Control (A) to MuH (G)] passed the normality test at α = 0.05, as their p-values (ranging from 0.1447 to 0.9531) exceeded the significance threshold, confirming no significant deviation from normality. The test statistic W (closer to 1 implies stronger normality) further supports this, with values like 0.9779 (Group E: CoH) suggesting near-ideal normality, while lower values (e.g., 0.9119 for Group D CiH) were still within acceptable limits. Notably, Group F (LyH; p = 0.2014) initially flagged in the D’Agostino-Pearson test was deemed normal by Shapiro-Wilk, highlighting its stricter sensitivity to non-normality in small samples. ns, not significant.

**Figure S13: Levene’s test (for homoscedasticity assessment) for Figure 6C.** The Levene’s test results in the Figure show that the deviation from zero is non-significant (p > 0.05) for all groups from Control (A) to MuH (G), indicating homoscedasticity (equal variances) across the datasets. This is beneficial for the experiment because it validates the assumption of equal variances required for reliable linear regression analysis, ensuring that the comparisons of slopes and intercepts are not biased by heteroscedasticity. The non-significant result supports the robustness of the regression models and justifies the use of standard parametric interpretations for the fitted lines.

**Figure S14: Quantitative analysis report of intracellular fatty acids in HepG2 cells under control conditions as determined by GC-MS-based chromatographic profiling.** The figure shows a representative sample chromatogram with retention time on the x-axis and signal intensity (counts) on the y-axis, followed by a summary table of the identified fatty acids, including their retention times, detector responses, and relative abundance (%). This report illustrates the separation and quantification performance of the analytical method for multiple saturated and unsaturated fatty acids within a single run, supporting the reliability of the fatty acid profiling employed in the study.

**Figure S15: Quantitative analysis report of intracellular fatty acids in HepG2 cells treated with palmitic acid as determined by GC-MS-based chromatographic profiling.** The figure shows a representative sample chromatogram with retention time on the x-axis and signal intensity (counts) on the y-axis, followed by a summary table of the identified fatty acids, including their retention times, detector responses, and relative abundance (%). This report illustrates the separation and quantification performance of the analytical method for multiple saturated and unsaturated fatty acids within a single run, supporting the reliability of the fatty acid profiling employed in the study.

**Figure S16: Quantitative analysis report of intracellular fatty acids in HepG2 cells treated with palmitic acid and metformin as determined by GC-MS-based chromatographic profiling.** The figure shows a representative sample chromatogram with retention time on the x-axis and signal intensity (counts) on the y-axis, followed by a summary table of the identified fatty acids, including their retention times, detector responses, and relative abundance (%). This report illustrates the separation and quantification performance of the analytical method for multiple saturated and unsaturated fatty acids within a single run, supporting the reliability of the fatty acid profiling employed in the study.

**Figure S17: Quantitative analysis report of intracellular fatty acids in HepG2 cells treated with palmitic acid and CiH as determined by GC-MS-based chromatographic profiling.** The figure shows a representative sample chromatogram with retention time on the x-axis and signal intensity (counts) on the y-axis, followed by a summary table of the identified fatty acids, including their retention times, detector responses, and relative abundance (%). This report illustrates the separation and quantification performance of the analytical method for multiple saturated and unsaturated fatty acids within a single run, supporting the reliability of the fatty acid profiling employed in the study.

**Figure S18: Quantitative analysis report of intracellular fatty acids in HepG2 cells treated with palmitic acid and CoH as determined by GC-MS-based chromatographic profiling.** The figure shows a representative sample chromatogram with retention time on the x-axis and signal intensity (counts) on the y-axis, followed by a summary table of the identified fatty acids, including their retention times, detector responses, and relative abundance (%). This report illustrates the separation and quantification performance of the analytical method for multiple saturated and unsaturated fatty acids within a single run, supporting the reliability of the fatty acid profiling employed in the study.

**Figure S19: Quantitative analysis report of intracellular fatty acids in HepG2 cells treated with palmitic acid and LyH as determined by GC-MS-based chromatographic profiling.** The figure shows a representative sample chromatogram with retention time on the x-axis and signal intensity (counts) on the y-axis, followed by a summary table of the identified fatty acids, including their retention times, detector responses, and relative abundance (%). This report illustrates the separation and quantification performance of the analytical method for multiple saturated and unsaturated fatty acids within a single run, supporting the reliability of the fatty acid profiling employed in the study.

**Figure S20: Quantitative analysis report of intracellular fatty acids in HepG2 cells treated with palmitic acid and MuH as determined by GC-MS-based chromatographic profiling.** The figure shows a representative sample chromatogram with retention time on the x-axis and signal intensity (counts) on the y-axis, followed by a summary table of the identified fatty acids, including their retention times, detector responses, and relative abundance (%). This report illustrates the separation and quantification performance of the analytical method for multiple saturated and unsaturated fatty acids within a single run, supporting the reliability of the fatty acid profiling employed in the study.
